# Encapsulated in silica: genome, proteome and physiology of the thermophilic bacterium *Anoxybacillus flavithermus *WK1

**DOI:** 10.1186/gb-2008-9-11-r161

**Published:** 2008-11-17

**Authors:** Jimmy H Saw, Bruce W Mountain, Lu Feng, Marina V Omelchenko, Shaobin Hou, Jennifer A Saito, Matthew B Stott, Dan Li, Guang Zhao, Junli Wu, Michael Y Galperin, Eugene V Koonin, Kira S Makarova, Yuri I Wolf, Daniel J Rigden, Peter F Dunfield, Lei Wang, Maqsudul Alam

**Affiliations:** 1 Department of Microbiology, University of Hawai'i, 2538 The Mall, Honolulu, HI 96822, USA; 2 GNS Science, Extremophile Research Group, 3352 Taupo, New Zealand; 3 TEDA School of Biological Sciences and Biotechnology, Nankai University, Tianjin 300457, PR China; 4 Tianjin Research Center for Functional Genomics and Biochip, Tianjin 300457, PR China; 5 Key Laboratory of Molecular Microbiology and Technology, Ministry of Education, Tianjin 300457, PR China; 6 National Center for Biotechnology Information, NLM, National Institutes of Health, Bethesda, MD 20894, USA; 7 Advance Studies in Genomics, Proteomics and Bioinformatics, College of Natural Sciences, University of Hawai'i, Honolulu, HI 96822, USA; 8 School of Biological Sciences, University of Liverpool, Crown Street, Liverpool L69 7ZB, UK; 9 Department of Biological Sciences, University of Calgary, 2500 University Dr. NW, Calgary, Alberta T2N 1N4, Canada; 10 Current address: Bioscience Division, Los Alamos National Laboratory, Los Alamos, NM 87545, USA

## Abstract

**Background:**

Gram-positive bacteria of the genus *Anoxybacillus *have been found in diverse thermophilic habitats, such as geothermal hot springs and manure, and in processed foods such as gelatin and milk powder. *Anoxybacillus flavithermus *is a facultatively anaerobic bacterium found in super-saturated silica solutions and in opaline silica sinter. The ability of *A. flavithermus *to grow in super-saturated silica solutions makes it an ideal subject to study the processes of sinter formation, which might be similar to the biomineralization processes that occurred at the dawn of life.

**Results:**

We report here the complete genome sequence of *A. flavithermus *strain WK1, isolated from the waste water drain at the Wairakei geothermal power station in New Zealand. It consists of a single chromosome of 2,846,746 base pairs and is predicted to encode 2,863 proteins. *In silico *genome analysis identified several enzymes that could be involved in silica adaptation and biofilm formation, and their predicted functions were experimentally validated *in vitro*. Proteomic analysis confirmed the regulation of biofilm-related proteins and crucial enzymes for the synthesis of long-chain polyamines as constituents of silica nanospheres.

**Conclusions:**

Microbial fossils preserved in silica and silica sinters are excellent objects for studying ancient life, a new paleobiological frontier. An integrated analysis of the *A. flavithermus *genome and proteome provides the first glimpse of metabolic adaptation during silicification and sinter formation. Comparative genome analysis suggests an extensive gene loss in the *Anoxybacillus*/*Geobacillus *branch after its divergence from other bacilli.

## Abstract 2

Sequencing of the complete genome of Anoxybacillus flavithermus reveals enzymes that are required for silica adaptation and biofilm formation.

## Background

Gram-positive bacteria of the genus *Anoxybacillus *were originally described as obligately anaerobic spore-forming bacilli. They are members of the family Bacillaceae, whose representatives were long believed to be obligate or facultative aerobes. However, it has been shown that *Bacillus subtilis *and several other bacilli are capable of anaerobic growth [1-3], whereas *Anoxybacillus *spp. turned out to be facultative anaerobes [4,5]. They are found in diverse moderate- to high-temperature habitats such as geothermal hot springs, manure, and processed foods such as gelatin [4,6,7]. *Anoxybacillus flavithermus *is a major contaminant of milk powder [8].

We report here the complete genome sequence of the thermophilic bacterium *A. flavithermus *strain WK1 [GenBank:CP000922], which was isolated from the waste water drain at the Wairakei geothermal power station in New Zealand [9]. This isolate has been deposited in Deutsche Sammlung von Mikroorganismen und Zellkulturen (DSMZ, Braunschweig, Germany) as strain DSM 21510. The 16S rRNA sequence of strain WK1 is 99.8% identical to that of the *A. flavithermus *type strain DSM 2641 [10], originally isolated from a hot spring in New Zealand [6]. The name '*flavithermus*' reflects the dark yellow color of its colonies, caused by accumulation of a carotenoid pigment in the cell membrane. *Anoxybacillus flavithermus*, formerly referred to as '*Bacillus flavothermus*', grows in an unusually wide range of temperatures, 30-72°C, and pH values, from 5.5 to 10.0 [6]. Temperature adaptation mechanisms in *A. flavithermus *proteins have attracted some attention to this organism [11]. However, a property of greater potential importance to the fields of paleobiology and astrobiology is its ability to grow in waters that are super-saturated with amorphous silica, and where opaline silica sinter is actively forming [9,12]. Flushed waste geothermal fluids from the Wairakei power station drain into a concrete channel at about 95°C. These fluids cool as they travel down the 2-km-long drainage channel, dropping to 55°C before entering Wairakei Stream. As the water cools down, silica sinter deposits subaqueously in the channels, forming precipitates composed of amorphous silica (opal-A) [9]. The ability of *A. flavithermus *to grow in super-saturated silica solutions makes it an ideal subject to study the processes of sinter formation, which might be similar to the biomineralization processes that occurred at the dawn of life [13]. Although bacteria are believed to play only a passive role in silicification, they definitely affect the absolute rate of silica precipitation by providing increased surface area. In addition, bacteria largely control the textural features of the resulting siliceous sinters [14]. We have obtained the complete genome sequence of *A. flavithermus *WK1 and employed it to analyze bacterial physiology and its changes in response to silica-rich conditions. This study sheds light on the biogeochemical processes that occur during the interaction between microbial cells and dissolved silica and result in sinter deposition.

## Results

### Genome organization

**Figure 1:**
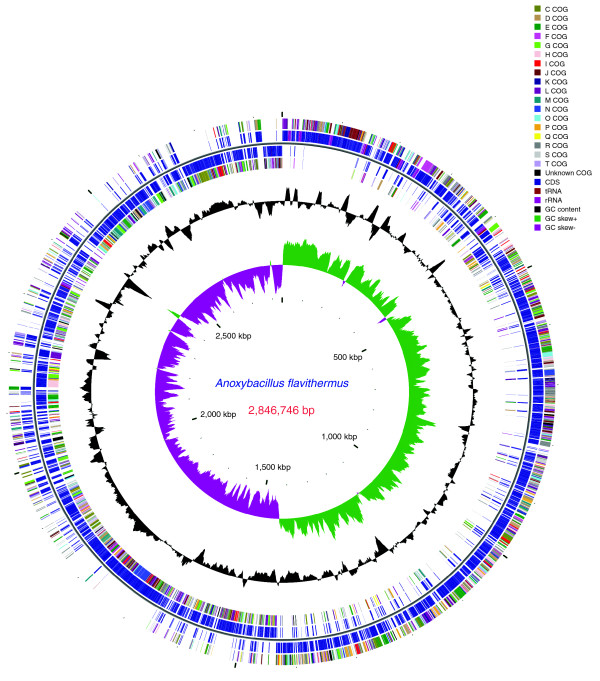
Circular representation of the *A. flavithermus *genome. The first and second circles show open reading frames (ORFs) in the positive strand: the first circle shows ORFs categorized by COG functional categories and the second circle shows coding sequences in blue and tRNA/rRNA genes in dark red. The third and fourth circles show ORFs in a similar fashion to the first and second circles but in the negative strand. The fifth circle shows variations in G+C content of the genome from the mean. The sixth circle shows a GC-skew plot of the genome showing approximate origin of replication and termination sites.

**Figure 2:**
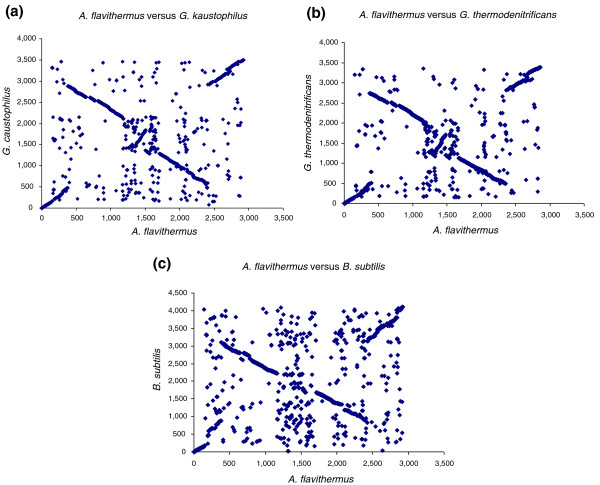
Pairwise genome alignments between **(a) ***A. flavithermus *and *G. kaustophilus*, **(b) ***A. flavithermus* and *G. thermodenitrificans*, and **(c) ***A. flavithermus *and *B. subtilis*. Each point indicates a pair of putative orthologous genes, identified as bidirectional best BLAST hits in the comparison of two proteomes.

The genome of *A. flavithermus *strain WK1 consists of a single, circular chromosome of 2,846,746 bp (Figure 1) with an average G+C content of 41.78% (Table 1). The genome encompasses 2,863 predicted protein-coding genes, 8 rRNA (16S-23S-5S) operons, 77 tRNA genes, and 19 predicted riboswitches. Of the 2,863 predicted proteins, 1,929 have been assigned probable biological functions, 418 were conserved proteins with only general function predicted, and for 516 putative proteins no function was predicted (of these, 110 proteins had no detectable homologs in the NCBI protein database). The genome contains one prophage region with 44 genes (Aflv_0639-0682) and encodes 105 transposases. In its gene order and the phylogenetic affinities of the encoded proteins, *A. flavithermus *WK1 is a typical member of the family Bacillaceae, with *Geobacillus kaustophilus *and *Geobacillus thermodenitrificans *as its closest neighbors (see below). Pair-wise genome alignments show high conservation of gene order between *A. flavithermus*, *G. kaustophilus *and *B. subtilis *(Figure 2). *Anoxybacillus flavithermus *WK1 has a typical firmicute proteome, with 89% of the predicted open reading frames (ORFs) having closest homologs in *Bacillus *spp. (Figure S1 in Additional data file 1). However, the *A. flavithermus *WK1 genome is the smallest among the sequenced members of Bacillaceae and generally encodes fewer paralogous proteins than other bacilli (Table S1 in Additional data file 1).

**Table 1:** Genome features of *A. flavithermus*

Genome size	2,846,746 bp
G+C content	41.78%
Number of predicted coding sequences	2,863, 104 RNA, 112 pseudogenes
Average size of coding sequences	860 bp
Percentage coding	90.2%
Number of protein coding genes	2,863 (22 with frame shifts)
Number of proteins with assigned biological function	1,929 (67%)
Number of proteins with predicted general function	418 (15%)
Number of proteins of unknown function	516 (18%)
Number of proteins assigned to COGs	2,526 (88%)
Number of tRNA genes	77
Number of rRNA operons	24
Number of small RNA genes	3
Number of riboswitches	19

### Metabolism

Despite its much smaller genome size, *A. flavithermus *appears to retain most of the key metabolic pathways present in *B. subtilis *and other bacilli. It has a complete set of enzymes for biosynthesis of all amino acids, nucleotides and cofactors, with the sole exception of the molybdenum cofactor (Table S2 in Additional data file 1). Cells of *A. flavithermus *had been originally reported to reduce nitrate [4,6]; however, in subsequent work, nitrate reductase activity has not been observed in this organism [15]. In accord with the latter report, the *A. flavithermus *WK1 genome encodes neither the assimilatory nitrate/nitrite reductase complex (NasBCDE) nor the respiratory nitrate reductase complex (NarGHJI), both of which are present and functional in *B. subtilis *[16,17], nor the third (proteobacterial) type of nitrate reductase (NapAB) [18]. Nitrate/nitrite transporters NasA and NarK are missing in *A. flavithermus *as well. The loss of nitrate reductases in *A. flavithermus *WK1 appears to be a recent event, given that *G. kaustophilus *encodes the assimilatory nitrate reductase, whereas *G. thermodenitrificans *encodes the respiratory nitrate reductase complex. In accordance with the loss of nitrate reductases, *A. flavithermus *WK1 has lost the entire set of enzymes involved in the biosynthesis of the molybdenum cofactor of nitrate reductase, as well as the molybdate-specific ABC (ATP-binding cassette)-type transporter, all of which are encoded in *G. kaustophilus *and *G. thermodenitrificans*. Molybdenum-dependent xanthine dehydrogenase and its homologs YoaE (putative formate dehydrogenase) and YyaE have been lost as well. As suggested in [19], the loss of molybdate metabolism could be part of a strategy to avoid generation of reactive oxygen species.

As the name suggests, members of the genus *Anoxybacillus *were initially described as obligate or facultative anaerobes [4,5]. However, the initial description of *(Anoxy)bacillus flavithermus *already mentioned its capability to grow in aerobic conditions [6]. Examination of the *A. flavithermus *WK1 genome revealed that it encodes an electron transfer chain that is as complex as that of *B. subtilis *and appears to be well-suited for using oxygen as terminal electron acceptor. The electron transfer chain of *A. flavithermus *includes NADH dehydrogenase, succinate dehydrogenase, quinol oxidases of *bd *type and *aa*_3 _type, menaquinol:cytochrome *c *oxidoreductase and cytochrome *c *oxidase, as well as two operons encoding the electron transfer flavoprotein (Table 2). *Anoxybacillus flavithermus *also encodes a variety of enzymes that are important for the defense against oxygen reactive species, such as catalase (peroxidase I), Mn-containing catalase, Mn-, Fe-, and Cu,Zn-dependent superoxide dismutases (the latter, in contrast to *B. subtilis *YojM, has both Cu-binding histidine residues), thiol peroxidase, and glutathione peroxidase (Table 2). The presence of these genes in the genome suggests that *A. flavithermus *WK1 should be able to thrive in aerobic conditions. Indeed, isolation of this strain, similarly to the type strain *A. flavithermus *DSM 2641, has been carried out in open air, without the use of anaerobic techniques [6,9,20].

**Table 2:** Electron transport and oxygen resistance genes of *A. flavithermus*

Genes	Locus tags	Functional annotation	*B. subtilis *orthologs
**Electron-transport chain**			
*nuoABCD HIJKLMN*	Aflv2700-Aflv2690	NADH dehydrogenase	-
*sdhCAB*	Aflv0580-Aflv0581	Succinate dehydrogenase	BSU28450-BSU28430
*cydAB*	Aflv0386-Aflv0385; Aflv0395-Aflv0394	Cytochrome *bd*-type quinol oxidase	BSU38760-BSU38750; BSU30710-BSU30720
*qoxABCD*	Aflv0272-Aflv0275	Cytochrome *aa*_3_-type quinol oxidase	
*etfBA*	Aflv0567-Aflv0568; Aflv1248-Aflv1249	Electron transfer flavoprotein	BSU28530-BSU28520
*qcrABC*	Aflv1113-Aflv1115	Menaquinol:cytochrome *c *oxidoreductase	BSU22560-BSU22540
*ctaCDEF*	Aflv1868-Aflv1865; Aflv1360-Aflv1359	Cytochrome *c *oxidase (*caa*_3_-type)	BSU14890-BSU14920
			
**Response to oxygen**			
*katG*	Aflv1200	Catalase (peroxidase I)	-
*yjqC*	Aflv1392	Mn-containing catalase	BSU12490
*sodA*	Aflv0876	Mn-superoxide dismutase	BSU25020
*sodF*	Aflv1031	Fe-superoxide dismutase	BSU19330
*yojM*	Aflv2392	Cu,Zn-superoxide dismutase	BSU19400
*tpx*	Aflv0478	Thiol peroxidase	BSU29490
*bsaA*	Aflv1322	Glutathione peroxidase,	BSU21900
*resABCDE*	Aflv1036_Aflv1040	Redox sensing and cytochrome biogenesis system	BSU23150-BSU23110

*Anoxybacillus flavithermus *WK1 grows well anaerobically in rich media, such as tryptic soy broth (TSB). Owing to the absence of nitrate and nitrite reductases (see above), its anaerobic growth cannot rely on nitrate or nitrite respiration and apparently proceeds by fermentation. Fermentative growth of *B. subtilis *requires phosphotransacetylase, acetate kinase and L-lactate dehydrogenase genes [1,3]. All these genes are conserved in *A. flavithermus *(*pta*, Aflv_2760; *ack*, Aflv_0480; *lctE*, Aflv_0889), suggesting that, like *B. subtilis*, this bacterium can ferment glucose and pyruvate into acetate [1]. However, catabolic acetolactate synthase AlsSD and acetolactate dehydrogenase, which are responsible for acetoin production by fermenting *B. subtilis *[1], are missing in *A. flavithermus*, indicating that it cannot produce acetoin.

In agreement with the experimental data [6], genome analysis indicates that *A. flavithermus *is able to utilize a variety of carbohydrates as sole carbon sources. It has at least four sugar phosphotransferase systems with predicted specificity for glucose, fructose, sucrose, and mannitol. Additionally, it encodes ABC-type transporters for ribose, glycerol-3-phosphate, and maltose, and several ABC-type sugar transporters of unknown specificity. A complete set of enzymes was identified for general carbohydrate metabolism (glycolysis, the TCA cycle, and the pentose phosphate pathway, but not the Entner-Doudoroff pathway). The *A. flavithermus *genome also contains a gene cluster (Aflv_2610-2618) that is very similar to the gene cluster associated with antibiotic production and secretion in many other Gram-positive bacteria [21], suggesting that *A. flavithermus *might be able to produce bactericidal peptides. It is not obvious which of these systems are relevant to the survival of *A. flavithermus *in silica solutions, but they might facilitate its growth in powdered milk and similar habitats.

### Evolution of the *Anoxybacillus *branch of bacilli

**Figure 3:**
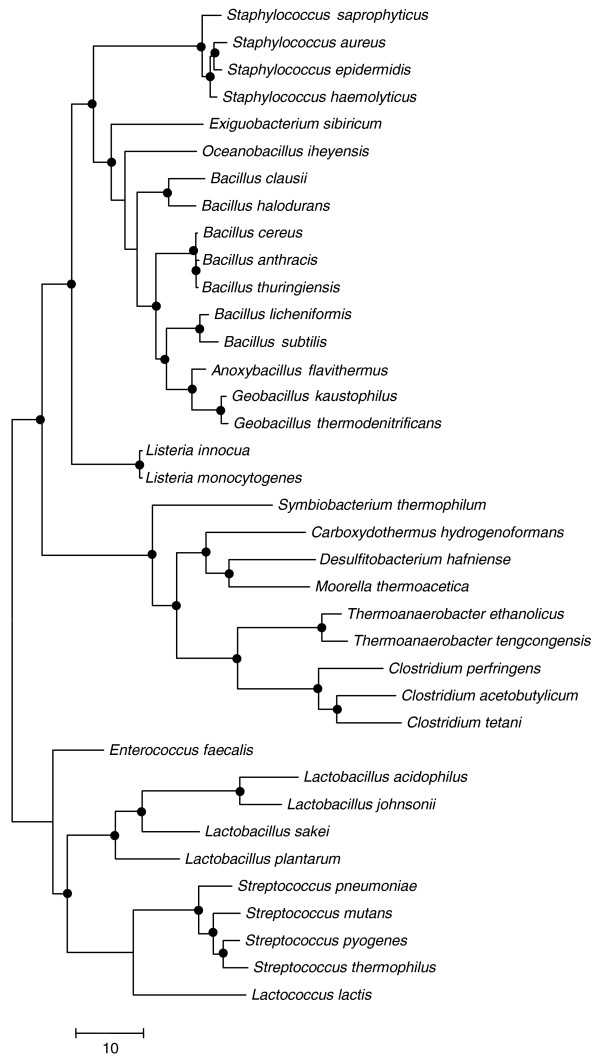
Phylogenetic tree of the Firmicutes based on concatenated sequences of RNA polymerase subunits RpoA, RpoB and RpoC. Branches that are supported by bootstrap probability >70% are marked by black circles.

In a phylogenetic tree constructed using a concatenated alignment of the RNA polymerase subunits RpoA, RpoB, and RpoC, *A. flavithermus*, *G. kaustophilus*, and *G. thermodenitrificans *grouped together and formed a deep branch within the *Bacillus *cluster (Figure 3). A distinct *Anoxybacillus*/*Geobacillus *branch is also seen in a gene content tree that was constructed on the basis of the presence or absence of particular protein families in the genomes of 26 species of firmicutes and 2 actinobacteria (used as an outgroup; Figure S2 in Additional data file 1).

**Figure 4:**
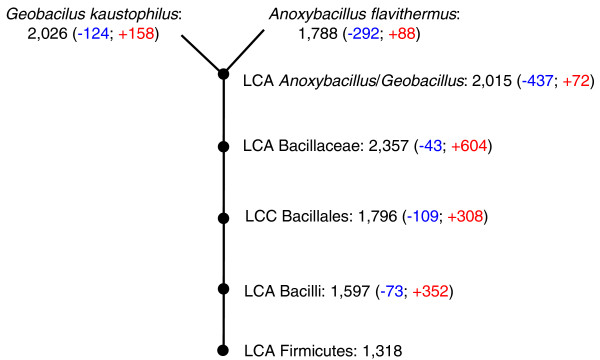
Predicted gene losses and gains in the evolution of the *Anoxybacillus *branch. The nodes (marked by black dots) indicate the last common ancestors (LCA) of the following taxonomic groups: the phylum Firmicutes, class Bacilli, order Bacillales, family Bacillaceae, and the *Anoxybacillus*/*Geobacillus *branch. Each node shows the predicted genome size of the given ancestral form and the likely number of gene losses and gains compared to the preceding node. The reconstruction of gene gains and losses was performed on the basis of COG phyletic patterns as described in [78].

*Anoxybacillus flavithermus *WK1 has a relatively small genome compared to other *Bacillus *species. To determine which genes were likely to have been lost and gained in this lineage, we reconstructed the most parsimonious scenario of evolution [22] from the last common ancestor of the firmicutes. The reconstruction was performed on the basis of the assignment of *A. flavithermus *to the Clusters of Orthologous Groups of proteins (COGs), followed by the comparison of COG-based phyletic patterns of 20 other bacilli, 5 clostridia, and 6 mollicutes. This approach assigned 2,015 genes (COGs) to the common ancestor of *A. flavithermus *and *G. kaustophilus *(Figure 4). The reconstruction results suggest that a massive gene loss (-437 genes) occurred during evolution from the common ancestor of Bacillaceae to the common ancestor of *Anoxybacillus *and *Geobacillus*. The majority of the genes shared between *A. flavithermus *and *G. kaustophilus *are also shared with other *Bacillus *species. Gene losses in the *Geobacillus*/*Anoxybacillus *branch include, among others, genes encoding the nitrogen regulatory protein PII, ABC-type proline/glycine betaine transport system, methionine synthase II (cobalamin-independent), sorbitol-specific phosphotransferase system, β-xylosidase, and some dTDP-sugar metabolism genes (Table S3 in Additional data file 1). However, 62 gene gains were inferred as well, including several genes coding for cobalamin biosynthesis enzymes, methylmalonyl-CoA mutase, genes involved in assembly of type IV pili (Aflv_0630-0632), an uncharacterized ABC-type transport system, and 16 genes encoding uncharacterized conserved proteins (Table S3 in Additional data file 1). After the split of the *Anoxybacillus *and *Geobacillus *lineages, *A. flavithermus *continued to show strong genome reduction (-292 genes) compared to *G. kaustophilus *(-124 genes), losing, in particular, some genes of nitrogen and carbohydrate metabolism. In addition, *A. flavithermus *has apparently experienced less gene gain (+88) than *G. kaustophilus *(+158). The few genes likely acquired in the *Anoxybacillus *lineage include the clustered regularly interspaced short palindromic repeat (CRISPR)-associated genes (Aflv_0764-0771) that form an antisense RNA-based system of phage resistance, which is often associated with thermophily [23,24].

### Signal transduction

Being a free-living environmental microorganism, *A. flavithermus *encodes numerous proteins involved in signal transduction. These include 23 sensor histidine kinases and 24 response regulators (16 pairs of which are clustered in operons), 20 methyl-accepting chemotaxis proteins, 5 predicted eukaryotic-type Ser/Thr protein kinases, and 21 proteins involved in metabolism of cyclic diguanylate (cyclic (3',5')-dimeric guanosine monophosphate (c-di-GMP)), a recently recognized secondary messenger that regulates transition from motility to sessility and biofilm formation in a variety of bacteria [25]. Compared to other bacilli, this set is significantly enriched in chemotaxis transducers and c-di-GMP-related proteins [26]. *Anoxybacillus flavithermus *encodes 12 proteins with the diguanylate cyclase (GGDEF) domain, 6 of which also contain the c-di-GMP phosphodiesterase (EAL) domain, and one combines GGDEF with an alternative c-di-GMP phosphodiesterase (HD-GYP) domain. *Anoxybacillus flavithermus *WK1 also encodes two proteins with the EAL domain and seven proteins with the HD-GYP domain that do not contain the GGDEF domain. In addition, it encodes two proteins with the PilZ domain [27], which serves as a c-di-GMP-binding adaptor protein [28,29]. The total number of proteins implicated in c-di-GMP turnover in *A. flavithermus *is third highest among all Gram-positive bacteria sequenced to date, after *Clostridium difficile *and *Desulfitobacterium hafniense*, which have much larger genomes [26,30].

### Silicification of *A. flavithermus *cells and biofilm formation

**Figure 5:**
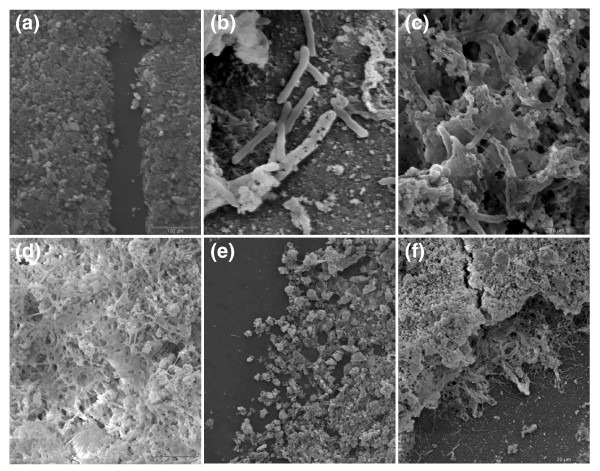
Role of *A. flavithermus *cells and biofilms in silica precipitation. **(a) **Subaqueous amorphous silica (opal-A) precipitated on glass substrate (dark gray). **(b) **Heavily silicified and unsilicified *A. flavithermus *cells showing a discontinuous sheath of uniform thickness surrounding one cell. **(c,d) **Association of silica precipitates with the extracellular matrix produced by biofilm-forming cells of *A. flavithermus*. **(e) ***A. flavithermus *biofilm with extensive granular silica precipitates. The glass substrate to the left shows little silica precipitation and would resemble (a) under high magnification. **(f) **Extensively silicified *A. flavithermus *biofilm showing variably silicified cells and a continuous outer coating of silica. Each plate represents a scanning electron microphotograph with scale bar as shown in the bottom right corner.

The abundance of c-di-GMP-related proteins suggests that regulation of biofilm formation plays an important role in the physiology of *A. flavithermus*. Indeed, scanning electron microphotographs of *A. flavithermus *cells cultured in the presence of high amounts of silica showed that the presence of biofilm had a major effect on the form of silica precipitation. In the absence of bacteria, the prevailing mode of silica precipitation was the formation of a layer of amorphous silica nanospherules (Figure 5a). In the presence of bacteria, silica precipitates were often associated with individual cells of *A. flavithermus *(Figure 5b), suggesting that these cells might serve as nucleation sites for sinter formation. However, in the culture of *A. flavithermus *cells attached as a biofilm to a glass slide, silica precipitates were mostly bound to the exopolysaccharide material of the biofilm (Figure 5c,d). Biofilm-associated silica was often seen forming extensive granular silica precipitates (Figure 5e). Further incubation led to the development of a complex, multi-layered biofilm that was impregnated with silica particles (Figure 5f). Obviously, *A. flavithermus *biofilm formation played a key role in determining the structural nature of the silica sinter. Indeed, *A. flavithermus *WK1 retains some of the genes (Table 3) that are required for biofilm formation in *B. subtilis *[31,32]. Proteins encoded by these genes include: the master regulators of biofilm formation AbrB (Aflv_0031) and SinR (Aflv_2245); α-phosphoglucomutase YhxB (Aflv_2333), which is probably involved in exopolysaccharide synthesis; EcsB (Aflv_2284), the membrane subunit of an ABC-type transporter that could promote secretion of protein components of the extracellular matrix; an HD-superfamily hydrolase YqeK (Aflv_0816) that is required for the formation of thick pellicles; YlbF (Aflv_1855), a positive regulator of competence factor ComK; and YmcA (Afla1522), a protein of unknown function. Other biofilm-forming proteins of *B subtilis*, namely, the AbrB- and SinR-regulated genes *tasA *(*yqhF*) or *yqfM *[33,34], are absent in the smaller genome of *A. flavithermus*.

**Table 3:** *A. flavithermus *orthologs of biofilm-related genes of *B. subtilis*

*B. subtilis*			*A. flavithermus*	
			
Gene	Locus tag	Functional annotation	Ortholog	COG number
*abrB*	BSU00370	Transcriptional regulator	Aflv_0031	COG2002
*pgcA *(*yhxB*)	BSU09310	Alpha-phosphoglucomutase	Aflv_2333	COG1109
*sipW*	BSU24630	Signal peptidase	-	COG0681
*yqxM*	BSU24640	Biofilm formation protein	-	-
*ecsB*	BSU10050	ABC transporter subunit	Aflv_2284	COG4473
*yqeK*	BSU25630	HD-superfamily hydrolase	Aflv_0816	COG1713
*ylbF*	BSU14990	Regulatory protein (regulator of ComK)	Aflv_1855	COG3679
*ymcA*	BSU17020	Unknown function	Aflv_1522	COG4550
*sinR*	BSU24610	Transcriptional regulator	Aflv_2245	COG1396
*tasA*	BSU24620	Camelysin, spore coat-associated metalloprotease	-	-
*yveQ*	BSU34310	Capsular polysaccharide biosynthesis protein EpsG	-	-
*yveR*	BSU34300	Capsular polysaccharide biosynthesis glycosyl transferase EpsH	Aflv_2196	COG0463

### Cell adaptation to silica

**Figure 6:**
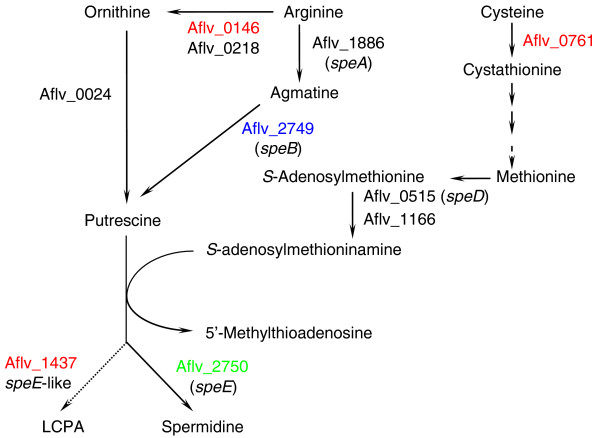
Proposed long-chain polyamine (LCPA) biosynthesis pathway in *A. flavithermus*. Enzymatic reactions are shown as arrows and labeled with *A. flavithermus *gene products, predicted to catalyze these reactions. Proteins detected on the two-dimensional gels are shown in color: those that were up-regulated after incubation for 8 hours in the presence of 10.7 mM silica are indicated in red; Aflv_2750, whose expression was down-regulated, is indicated in green; blue color indicates proteins whose expression remained unchanged; and black color indicates proteins that were not detected on the two-dimensional gels. The functions of Aflv_0146 as arginase, Aflv_1886 as arginine decarboxylase, Aflv_0024 as ornithine decarboxylase, Aflv_2749 as agmatinase, Aflv_2750 as spermidine synthase, and Aflv_1437 as spermine synthase have been biochemically confirmed.

The existence of c-di-GMP-mediated signal transduction pathways also suggested that biofilm formation in *A. flavithermus *could be regulated in response to environmental conditions. To investigate possible mechanisms of silica adaptation, we compared protein expression profiles of *A. flavithermus *in the presence and absence of silica using two-dimensional electrophoresis and matrix-assisted laser desorption/ionization-time of flight (MALDI-TOF) mass spectrometry analyses (Figure S3 in Additional data file 1). Although samples from three independent experiments showed significant variance and the expression changes could not be statistically proven (Table S4 in Additional data file 1), the trends that they revealed provided certain clues to the *A. flavithermus *adaptation to silica. After exposure of batch cultures to 10.7 mM (300 ppm) silica (a mixture of monomeric H_4_SiO_4 _and polymerized silicic acid [35]) for 8 hours, expression of 19 proteins was increased at least 1.5-fold in each of three independent experiments, whereas expression of 18 proteins was found to be decreased (Table S4 in Additional data file 1). Most of these proteins were products of house-keeping genes whose up- or down-regulation could be related to the general stress in the presence of silica, as suggested by the increased expression of the alkaline shock protein Asp23 (Aflv_1780) and the carboxylesterase YvaK (Aflv_2499), which are stress-induced in *B. subtilis *[36]. The increased expression of AbrB (Aflv_0031), a key transcriptional regulator of biofilm-related genes in *B. subtilis*, suggested that exposure to silica could, indeed, trigger biofilm formation by *A. flavithermus*. Of particular interest was the differential effect of silica on the expression of two close paralogs, putrescine aminopropyltransferase (spermidine synthase) SpeE (Aflv_2750) and SpeE-like protein Aflv_1437. Expression of SpeE, which is part of the polyamine biosynthesis pathway of *B. subtilis *[37], was suppressed by exposure to silica. In contrast, SpeE-like protein Aflv_1437, which could participate in the synthesis of some other polyamine(s) (see, for example, [38]), was up-regulated (Table S4 in Additional data file 1). A predicted arginase (Aflv_0146), which catalyzes the first step in the synthesis of putrescine (the substrate of SpeE), namely, conversion of arginine to ornithine (Figure 6), was also up-regulated, whereas the expression of predicted arginine decarboxylase (Aflv_1886) and agmatinase (Aflv_2749), which comprise an alternative route for the synthesis of putrescine, was very low and, apparently, remained unchanged (data not shown), suggesting that putrescine was primarily produced via the arginase route. Given that long-chain polyamines (LCPAs) are crucial in the formation of silica nanostructures in diatoms [39-43], these data suggested a link between polyamine biosynthesis and biofilm formation in *A. flavithermus*. As a first step towards characterizing this link, proteins encoded by genes Aflv_0024, Aflv_0146, Aflv_1437, Aflv_1886, Aflv_2749, and Aflv_2750 were individually expressed, purified, and confirmed to function as, respectively, ornithine decarboxylase, arginase, spermine synthase, arginine decarboxylase, agmatinase, and spermidine synthase (Figures S4-S6 in Additional data file 1). In the general route, spermine synthase converts spermidine into spermine by transferring an aminopropyl group. The spermine synthase (Aflv_1437) identified here converts putrescine directly into spermine by adding two aminopropyl groups, raising the possibility of the formation of longer chain polyamines by sequentially adding multiple aminopropyl groups. The proposed roles of these enzymes in LCPA biosynthesis in *A. flavithermus *are shown in Figure 2.

We also examined protein expression profiles in the cells grown in the presence or absence of silica for 7 days. Sinters started forming in the silica-containing sample 5 days after inoculation, so by the end of the incubation the cells became silicified. Owing to the problems with collecting and analyzing silicified *A. flavithermus *cells, no attempt has been made to replicate this experiment, so these results were only considered in comparison to the samples from 8-hour exposure to silica. Spermine synthase Aflv_1437 was not detected in either silicified or control cells (last column of Table S4 in Additional data file 1), and arginase (Aflv_0146; Figure S7 in Additional data file 1) was only detected in the silicified cells at very low abundance. In contrast, spermidine synthase Aflv_2750 was detected at similar levels in both types of cells, indicating general cellular functions for spermidine. Remarkably, the transcriptional regulator AbrB (Aflv_0031) remained moderately up-regulated in the silicified cells, suggesting that it might play a general role in silica adaptation of *A. flavithermus*. Also up-regulated in both silica conditions were chemotaxis response regulator CheY (Aflv_1727), thiol peroxidase Aflv_0478, which is apparently involved in antioxidant defense, and methionine aminopeptidase Aflv_0127. Those proteins could also play a role in silica niche adaptation of *A. flavithermus*.

## Discussion

Silica precipitation and formation of sinter is an important geochemical process in hot spring systems, and understanding how these structures form might be important for deciphering some of the earliest biological processes on Earth [13,14].

Microbial fossils are well preserved in silica compared to CaCO_3 _or iron precipitates [13], and silica sinters are excellent structures for studying ancient microbial life. Microorganisms were previously believed to play no active role in the formation of silica precipitates. Rather, microbial cell surfaces have been assumed to provide nucleation sites to allow precipitation of minerals [14]. However, several recent studies have shed light on the biotic components that might play an active role in silicification. The best studied in this respect are diatoms, which build silica nanostructures in a controlled manner and under ambient conditions [44,45]. Formation of silica nanostructures in diatoms is influenced by polycationic peptides, named silaffins [39,46], and LCPAs [47]. In diatom cells, silica is deposited as nanospheres before being transformed into complex structures [48,49]. Polyamines have been shown to catalyze siloxane-bond formation and can also act as flocculating agents, leading to silica polymerization [50,51]. In the bacterial world, polyamines have been shown to be essential for biofilm formation in *Yersinia pestis *[52] and to activate biofilm formation in *Vibrio cholerae*, although, in the latter case, the effect appeared to be due primarily to intracellular signaling [53]. Studies of silicate binding by *B. subtilis *cell walls by Terry Beveridge and colleagues showed that it was electrostatic in nature and depended on the surface charge [54,55]. The observations of silica nanospheres formed around the bacterial cells in hot springs [9] and in simulated experimental conditions with *A. flavithermus *(Figure 5e) suggest that silica formation in hot springs also might be biologically influenced.

LCPAs participate in silica formation in diatoms [40-42] and enzymes similar to spermidine and spermine synthases are thought to be required for their synthesis [56]. On the other hand, polyamines, including putrescine, spermidine, and spermine, are ubiquitous in all cells, and play essential roles in cell proliferation and differentiation [57,58]. Of the two *speE *paralogs in *A. flavithermus *WK1, SpeE (Aflv_2750) catalyzes the formation of spermidine from putrescine, most likely for general cellular functions, whereas the SpeE-like Aflv_1437 catalyzes the conversion of putrescine into spermine and could be an important part of LCPA production. In *B. subtilis*, polyamines are synthesized via a single route, the agmatine pathway encoded by *speA *and the *speEB *operon [34]. Enzymes for this route are also encoded in *A. flavithermus *and most likely serve normal cellular functions as the expression level of arginine decarboxylase (Aflv_1886), the key enzyme of the pathway, was not stimulated by silica. Therefore, up-regulation of putrescine production for SpeE-like production was through the other route catalyzed by arginase and ornithine decarboxylase. The presence of two putrescine synthesis routes and two putrescine aminopropyltransferase homologs (SpeE and SpeE-like) indicates that polyamine synthesis is crucial for the specific niche adaptation of *A. flavithermus*.

Based on the proposed LCPA synthesis pathway (Figure 6), conversion of putrescine into spermine by the SpeE-like protein Aflv_1437 could be followed by further transfer of aminopropyl groups leading to the formation of LCPAs. Previous studies using computer simulations have shown that polyamine chains may self-assemble into structures serving as scaffolding or nucleation sites for the precipitation of silica-polyamine complexes [41]. Our results suggest that the SpeE-like enzyme may be responsible for the production of LCPAs that form the basis or scaffolding needed for the silica-polyamine complexes to aggregate.

Biofilm formation and production of exopolysaccharides are important processes that could facilitate silica sinter formation in hot springs. The abundance of c-di-GMP-related proteins in the *A. flavithermus *genome, as well as the up-regulation of the global regulator AbrB (Aflv_0031) in the presence of silica, suggests that biofilm formation by this organism is part of its global response to silica. In studies of the cyanobacterium *Calothrix *sp., silicification had no significant effect on cell viability [59]; there is little doubt that *A. flavithermus *cells remain viable during silicification as well. Our current working model implies that polymerization of monomeric and polymeric silica into silica nanospheres is facilitated by biotic factors such as LCPAs, as indicated by our proteomics results. Attachment of these silica nanospheres to the exopolysaccharide coating surrounding the *A. flavithermus *cells (Figure 5e) is a key step in silica sinter formation. In summary, this integrated genomics and proteomics study provides the first experimental evidence of the biochemical reactions between dissolved silica and the bacterial cell. Such reactions are likely to be crucial in the preservation of ancient microbial life and the growth of modern hot spring sinter deposits.

## Conclusion

The complete genome sequence of *A. flavithermus *shows clear signs of genome compaction in the *Anoxybacilus*/*Geobacillus *branch, compared to other members of the family Bacillaceae. In *A. flavithermus *strain WK1, adaptations to growth at high temperatures in supersaturated silica solutions include general streamlining of the genome, coupled with preservation of the major metabolic pathways and the capability to form biofilms. The presence of bacteria appears to affect silicification in several different ways. Passive effects of bacteria include providing nucleation sites for sinter formation and an increased surface area for silica precipitation. In addition, synthesis of LCPAs and biofilm formation by *A. flavithermus *could regulate sinter formation and control the textural features of the resulting siliceous sinters. The presence of an array of c-di-GMP-related signal transduction proteins suggests that *A. flavithermus *could regulate biofilm formation in response to the environmental conditions.

## Materials and methods

### Sequencing, assembly, and annotation

The genome of *A. flavithermus *was sequenced using the whole-genome-shotgun approach as previously described [60], using genomic DNA that was randomly sheared to generate 3 kb and 6 kb fragments. These fragments were size-selected on agarose gels, purified, end-repaired, ligated to pUC118 vectors, and transformed into DH10B competent cells by electroporation. Plasmids from positive clones were sequenced using Beckman CEQ 8000 (Beckman Coulter, Fullerton, CA, USA) and ABI 3730xl (Applied Biosystems, Foster City, CA, USA) sequencers. A total of 55,975 valid sequences were used for assembly with PHRED/PHRAP/CONSED [61], CAP3 [62], and SEQMAN II (DNAStar) programs. Further 3,863 sequences were used to close gaps between contigs and to improve overall sequence quality of contigs. Long PCR reactions were performed to verify sequence assembly. Protein-coding genes were predicted using GLIMMER [63] followed by BLASTX [64] searches of intergenic regions between predicted ORFs. Transfer RNAs were predicted by tRNAscan-SE [65]. Genome annotation was performed by running BLAST and PSI-BLAST against the NCBI protein database and the COG database with manual verification as described previously [60]. Metabolic pathways were analyzed by comparing COG assignments of *A. flavithermus *proteins with the standard sets of COGs involved in each pathway [66]. Phylogenetic analysis was performed as described [67].

### Biofilm formation and silica precipitation

Biofilm formation by *A. flavithermus *cells grown in the presence of silica was studied by incubating the cells in a chemostat-like system, consisting of a 500 ml serum vial, capped with a rubber seal with two input and one output lines. This was filled halfway with 300 ml of TSB. The two input lines were fed through the rubber seal and connected via peristaltic pumps to sterile reservoirs. One reservoir contained 2 × TSB and the other water. They were each fed at 0.15 ml per minute giving 1 × TSB in the vial. An output line connected to another peristaltic pump maintained the medium level at 300 ml. A final output line was fit with a luer valve and syringe to allow samples to be removed from the reservoir. A glass slide stood upright in the vial as a substrate for silica sinters, to be observed by scanning electron microscopy at the conclusion of the experiment. The cultivation vessel was contained in a 60°C oven and shaken gently at 100 rpm to simulate wave motion. After running the system for two days to ensure it was sterile, the medium was inoculated with *A. flavithermus *WK1 through the luer-fitted line. The system ran for two days to build up cell mass, then samples of 200 ml were taken for three successive days. Samples were centrifuged, the pellet washed 3 times in buffer (68 mM NaCl, 3 mM KCl, 1.5 mM KH_2_PO_4_, 9 mM NaH_2_PO_4_, 50 mM TRIS, pH 8.0), stored at -20°C and later freeze-dried. The system was running at pH 5.8 and OD_600 _0.15 during this time. After three days, the water was replaced with 1,000 mg/kg silica solution adjusted to pH 7. This flowed through a 200°C oven before reaching the cultivation vessel in order to monomerize the silica and sterilize the water. Samples were taken after 1, 2, 3 and 7 days and prepared as above. As time progressed, there was increasingly more solid, amorphous silica in the vessel, as this was not removed by the outflow. The system remained at pH 5.8 but there was no longer any way to reliably measure OD_600 _because of the silica precipitate. At the end of incubation, some of the amorphous silica and the slide were removed to be fixed in 2% glutaraldehyde. Samples for scanning electron microscopy were removed from storage and allowed to air-dry before coating with gold/palladium. Scanning electron microscopy examination was done on a Hitachi S-800 Field Emission scanning electron microscope operating at 15 kV.

### Proteomic analysis

*Anoxybacillus flavithermus *cells were grown in TSB at 60°C with shaking at 200 rpm on an orbital shaker (Thermo Electron Co., Waltham, MA, USA) to OD_600 _of 0.6, followed by the addition of silica to a final concentration of 10.7 mM and growth for another 8 hours. The same batch of the culture without added silica served as the control. Cells were harvested by centrifugation at 10,000 × g at 4°C for 10 minutes, extracellular proteins from the supernatant were collected and cellular proteins from the pellet were solubilized [68]. Immunoelectrophoresis (the first dimension) was carried out on IPG strips (Amersham Pharmacia Biotech, Uppsala, Sweden) in a Multiphor II electrophoresis unit (Amersham Pharmacia Biotech) with running conditions as described by Büttner *et al*. [69]. For the second dimension, vertical slab SDS-PAGE (12%) was run in a Bio-Rad Protean II Xi unit (Bio-Rad Laboratories, Hercules, CA, USA). Gels were stained with colloidal CBB G-250 [70], and scanned with a PowerLook 1000 (UMAX Technologies Inc., Fremont, CA, USA). PDQuest version 7.3.0 (Bio-Rad) was used for image analysis. Proteins were classified as being differentially expressed under the two conditions when spot intensity showed at least 1.5-fold change.

For protein identification, spots were excised from the gels, washed with 25 mM NH_4_HCO_3 _in 50% (v/v) acetonitrile for 3 × 15 minutes at room temperature, dried in a vacuum centrifuge, and incubated in 50 μl digestion solution consisted of 25 mM NH_4_HCO_3 _in 0.1% acetic acid and 12.5 ng/mL of trypsin (Promega, Madison, WI, USA) at 37°C overnight. The digested protein (0.3 μl) was spotted on a MALDI sample plate with the same volume of matrix (10 mg/ml α-cyano-4-hydroxycinnamic acid in 50% acetonitrile, 0.1% trifluoroacetic acid). Peptide mass spectra were obtained on a MALDI-TOF/TOF mass spectrometer (4700 Proteomics Analyzer, Applied Biosystems) in the positive ion reflector mode. The mass spectrometry spectra were internally calibrated with a mass standard kit for the 4700 Proteomics Analyzer. Proteins were identified by automated peptide mass fingerprinting using the Global Proteome Server Explorer™ software (Version 3.5, Applied Biosystems) against an in-house sequence database of *A. flavithermus *proteins. Peak lists (S/N > 10) were extracted from raw data for the data processing, and positive identifications were accepted up to 95% of confidence level. The following criteria were used for the database searches: maximum one missed cleavage per peptide; mass tolerance of 0.1 Da, and the acceptation of carbamidomethylation for cysteine and oxidation for methionine.

### Characterization of enzymes involved in LCPA synthesis

The genes Aflv_0024, Aflv_1886, Aflv_2749, and Aflv_1437 were cloned into the pET-14b vector, Aflv_0146 into pET-3a, and Aflv_2750 into pET-28a. *Escherichia coli *strain BL21 carrying each of the recombinant plasmids was grown overnight with shaking at 37°C in Luria broth containing 100 mg/ml ampicillin. The overnight culture (4 ml) was inoculated into 400 ml of fresh Luria broth and grown to mid-log phase (A_600 _= 0.6). Expression of Aflv_0146, Aflv_1437, Aflv_1886, Aflv_2749, and Aflv_2750 products was induced with 0.1 mM isopropyl β-D-1-thiogalactopyranoside (IPTG) at 37°C for 4 hours, and expression of the Aflv_0024 product was induced with 0.1 mM IPTG at 12°C for 8 hours. After IPTG induction, the cells were harvested by centrifugation at 6,000 × g at 4°C for 5 minutes, washed with binding buffer (10 mM imidazole, 300 mM NaCl and 50 mM Tris-HCl, pH 8.0), resuspended in 5 ml of binding buffer containing 1 mM phenylmethylsulfonyl fluoride and 1 mg/ml of lysozyme, and sonicated for 10 1-minute cycles with 1-second pulse on alternating 1-second pulse off at 95% of the maximum power (200 W) using an UP200S Ultraschallprozessor with a tapered microtip. The lysate of Aflv_1886 was further incubated for 10 minutes at 60°C. After centrifugation at 12,000 × g at 4°C for 30 minutes, the crude extract containing 6× His-tagged fusion proteins was purified by nickel ion affinity chromatography with a Chelating Sepharose Fast Flow column (GE Healthcare, Piscataway, NJ, USA) according to the manufacturer's instructions. The column was washed successively with 100 ml of wash buffer (25 mM imidazole, 300 mM NaCl, and 50 mM Tris-HCl, pH 8.0), and the fusion proteins were eluted with the elution buffer (250 mM imidazole, 300 mM NaCl and 50 mM Tris-HCl, pH 8.0), and dialyzed in 0.1 M Tris-HCl buffer (pH 8.8). Protein concentration was determined by the Bradford method. For SDS-PAGE, proteins were denatured at 100°C for 5 minutes in the presence of 0.1% SDS and 1% 2-mercaptoethanol, loaded in a 5% (w/v) stacking gel and separated in a 10% (w/v) separation gel. The gel was stained with Coomassie Bright Blue R250. The molecular weight markers were from the LMW-SDS Marker Kit (GE Healthcare).

Reactions catalyzed by arginase, arginine decarboxylase, ornithine decarboxylase, agmatinase, spermidine synthase and spermine synthase were carried out as previously described [71-74]. The activities of arginase and arginine decarboxylase were determined by thin-layer chromatography [75]. The activities of the other enzymes were assayed by high-performance liquid chromatography (HPLC) after Schotten-Baumann benzoylation as previously described [74,76,77]. HPLC analysis was performed with a Venusil XBP C18 column (4.6 × 250 mm) in conjunction with a LC-20AT (Shimadzu, Kyoto, Japan) HPLC apparatus. Benzoyl putrescine, spermidine and spermine were eluted by a gradient started with 60% methanol in water and proceeded linearly to 100% methanol, with a flow rate of 0.8 ml/minute over 20 minutes, and detected at a wavelength of 229 nm.

## Abbreviations

ABC: ATP-binding cassette; c-di-GMP: cyclic (3',5')-dimeric guanosine monophosphate; COGs: clusters of orthologous groups of proteins; HPLC: high-performance liquid chromatography; LCPA: Long-chain polyamine; MALDI-TOF: matrix-assisted laser desorption/ionization-time of flight; ORF: open reading frame; TSB: tryptic soy broth.

## Authors' contributions

BWM, PFD, LW, and MA designed the study. JHS, SH, and JAS performed genome sequencing. JHS, MVO, MYG, EVK, KSM, YIW, DJR and MA performed genome analysis. BWM, LF, MBS, DL, GZ, JW, PDF, and LW performed enzymatic and proteomic analysis. JHS, BWM, LF, MVO, MYG, EVK, KSM, DJR, PFD, LW and MA wrote the paper.

## Additional data files

The following additional data are available. Additional data file 1 contains Figures S1-S7 and Tables S1-S4.

## Supplementary Material

Additional data file 1 Figure S1: phylogenetic distribution of the best BLAST hits of *A. flavithermus *proteins. Figure S2: the tree of the phylum Firmicutes based on similarity of the phyletic patterns in COGs. Figure S3: two-dimensional gels comparing expression of *A. flavithermus *proteins from cells grown with or without silica. Figure S4: SDS-PAGE analysis of purified recombinant proteins. Figure S5: thin-layer chromatography-based detection of Aflv_0146 and Aflv_1886 reaction products ornithine and agmatine. Figure S6: HPLC chromatographs showing enzymatic activities of expressed Aflv_0024, Aflv_2749, Aflv_1437 and Aflv_2750 proteins. Figure S7: sequence alignment of *A. flavithermus *agmatinase Aflv_2749 and arginase Aflv_0146 with various agmatinases and arginases. Table S1: examples of paralogous proteins encoded in the genomes of *A. flavithermus *and five other bacilli. Table S2: presence or absence of certain metabolic pathway genes in the *A. flavithermus *genome. Table S3: examples of gene gains and losses in *Geobacillus*/*Anoxybacillus *lineages. Table S4: *A. flavithermus *genes that were found to be up- and down-regulated in cells exposed to silica. Click here for file

## References

[B1] Nakano MM, Dailly YP, Zuber P, Clark DP (1997). Characterization of anaerobic fermentative growth of *Bacillus subtilis*: identification of fermentation end products and genes required for growth.. J Bacteriol.

[B2] Nakano MM, Zuber P (1998). Anaerobic growth of a "strict aerobe" (*Bacillus subtilis*).. Annu Rev Microbiol.

[B3] Cruz Ramos H, Hoffmann T, Marino M, Nedjari H, Presecan-Siedel E, Dreesen O, Glaser P, Jahn D (2000). Fermentative metabolism of *Bacillus subtilis*: physiology and regulation of gene expression.. J Bacteriol.

[B4] Pikuta E, Lysenko A, Chuvilskaya N, Mendrock U, Hippe H, Suzina N, Nikitin D, Osipov G, Laurinavichius K (2000). *Anoxybacillus pushchinensis *gen. nov., sp. nov., a novel anaerobic, alkaliphilic, moderately thermophilic bacterium from manure, and description of *Anoxybacillus flavitherms *comb. nov.. Int J Syst Evol Microbiol.

[B5] Pikuta E, Cleland D, Tang J (2003). Aerobic growth of *Anoxybacillus pushchinoensis *K1^T^: emended descriptions of *A. pushchinoensis *and the genus *Anoxybacillus*.. Int J Syst Evol Microbiol.

[B6] Heinen W, Lauwers AM, Mulders JW (1982). *Bacillus flavothermus*, a newly isolated facultative thermophile.. Antonie van Leeuwenhoek.

[B7] De Clerck E, Vanhoutte T, Hebb T, Geerinck J, Devos J, De Vos P (2004). Isolation, characterization, and identification of bacterial contaminants in semifinal gelatin extracts.. Appl Environ Microbiol.

[B8] Rueckert A, Ronimus RS, Morgan HW (2005). Development of a rapid detection and enumeration method for thermophilic bacilli in milk powders.. J Microbiol Methods.

[B9] Mountain BW, Benning LG, Boerema JA (2003). Experimental studies on New Zealand hot spring sinters: rates of growth and textural development.. Can J Earth Sci.

[B10] Rainey FA, Fritze D, Stackebrandt E (1994). The phylogenetic diversity of thermophilic members of the genus *Bacillus *as revealed by 16S rDNA analysis.. FEMS Microbiol Lett.

[B11] Lauwers AM, Heinen W (1983). Thermal properties of enzymes from *Bacillus flavothermus*, grown between 34 and 70°C.. Antonie van Leeuwenhoek.

[B12] Pancost RD, Pressley S, Coleman JM, Benning LG, Mountain BW (2005). Lipid biomolecules in silica sinters: indicators of microbial biodiversity.. Environ Microbiol.

[B13] Konhauser KO, Jones B, Reysenbach AL, Renaut RW (2003). Hot spring sinters: keys to understanding Earth's earliest life forms.. Can J Earth Sci.

[B14] Konhauser KO, Jones B, Phoenix VR, Ferris G, Renaut RW (2004). The microbial role in hot spring silicification.. Ambio.

[B15] Belduz AO, Dulger S, Demirbag Z (2003). *Anoxybacillus gonensis *sp. nov., a moderately thermophilic, xylose-utilizing, endospore-forming bacterium.. Int J Syst Evol Microbiol.

[B16] Hoffmann T, Troup B, Szabo A, Hungerer C, Jahn D (1995). The anaerobic life of *Bacillus subtilis*: cloning of the genes encoding the respiratory nitrate reductase system.. FEMS Microbiol Lett.

[B17] Ogawa K, Akagawa E, Yamane K, Sun ZW, LaCelle M, Zuber P, Nakano MM (1995). The *nasB *operon and *nasA *gene are required for nitrate/nitrite assimilation in *Bacillus subtilis*.. J Bacteriol.

[B18] Richardson DJ, Berks BC, Russell DA, Spiro S, Taylor CJ (2001). Functional, biochemical and genetic diversity of prokaryotic nitrate reductases.. Cell Mol Life Sci.

[B19] Médigue C, Krin E, Pascal G, Barbe V, Bernsel A, Bertin PN, Cheung F, Cruveiller S, D'Amico S, Duilio A, Fang G, Feller G, Ho C, Mangenot S, Marino G, Nilsson J, Parrilli E, Rocha EP, Rouy Z, Sekowska A, Tutino ML, Vallenet D, von Heijne G, Danchin A (2005). Coping with cold: the genome of the versatile marine Antarctica bacterium *Pseudoalteromonas haloplanktis *TAC125.. Genome Res.

[B20] Burnett PG, Heinrich H, Peak D, Bremer PJ, McQuillan AJ, Daughney CJ (2006). The effect of pH and ionic strength on proton adsorption by the thermophilic bacterium *Anoxybacillus flavithermus*.. Geochim Cosmochim Acta.

[B21] Siezen RJ, Kuipers OP, de Vos WM (1996). Comparison of lantibiotic gene clusters and encoded proteins.. Antonie van Leeuwenhoek.

[B22] Mirkin BG, Fenner TI, Galperin MY, Koonin EV (2003). Algorithms for computing parsimonious evolutionary scenarios for genome evolution, the last universal common ancestor and dominance of horizontal gene transfer in the evolution of prokaryotes.. BMC Evol Biol.

[B23] Makarova KS, Grishin NV, Shabalina SA, Wolf YI, Koonin EV (2006). A putative RNA-interference-based immune system in prokaryotes: computational analysis of the predicted enzymatic machinery, functional analogies with eukaryotic RNAi, and hypothetical mechanisms of action.. Biol Direct.

[B24] Barrangou R, Fremaux C, Deveau H, Richards M, Boyaval P, Moineau S, Romero DA, Horvath P (2007). CRISPR provides acquired resistance against viruses in prokaryotes.. Science.

[B25] Römling U, Gomelsky M, Galperin MY (2005). C-di-GMP: The dawning of a novel bacterial signalling system.. Mol Microbiol.

[B26] Galperin MY (2005). A census of membrane-bound and intracellular signal transduction proteins in bacteria: bacterial IQ, extroverts and introverts.. BMC Microbiol.

[B27] Amikam D, Galperin MY (2006). PilZ domain is part of the bacterial c-di-GMP binding protein.. Bioinformatics.

[B28] Ryjenkov DA, Simm R, Römling U, Gomelsky M (2006). The PilZ domain is a receptor for the second messenger c-di-GMP: the PilZ domain protein YcgR controls motility in enterobacteria.. J Biol Chem.

[B29] Pratt JT, Tamayo R, Tischler AD, Camilli A (2007). PilZ domain proteins bind cyclic diguanylate and regulate diverse processes in *Vibrio cholerae*.. J Biol Chem.

[B30] Ulrich LE, Zhulin IB (2007). MiST: a microbial signal transduction database.. Nucleic Acids Res.

[B31] Branda SS, Gonzalez-Pastor JE, Dervyn E, Ehrlich SD, Losick R, Kolter R (2004). Genes involved in formation of structured multicellular communities by *Bacillus subtilis*.. J Bacteriol.

[B32] Hamon MA, Stanley NR, Britton RA, Grossman AD, Lazazzera BA (2004). Identification of AbrB-regulated genes involved in biofilm formation by *Bacillus subtilis*.. Mol Microbiol.

[B33] Chu F, Kearns DB, Branda SS, Kolter R, Losick R (2006). Targets of the master regulator of biofilm formation in *Bacillus subtilis*.. Mol Microbiol.

[B34] Branda SS, Chu F, Kearns DB, Losick R, Kolter R (2006). A major protein component of the *Bacillus subtilis *biofilm matrix.. Mol Microbiol.

[B35] Benning LG, Phoenix VR, Yee N, Tobin MJ (2004). Molecular characterization of cyanobacterial silicification using synchrotron infrared micro-spectroscopy.. Geochim Cosmochim Acta.

[B36] Hecker M, Volker U (2001). General stress response of *Bacillus subtilis *and other bacteria.. Adv Microb Physiol.

[B37] Sekowska A, Bertin P, Danchin A (1998). Characterization of polyamine synthesis pathway in *Bacillus subtilis *168.. Mol Microbiol.

[B38] Knott JM, Romer P, Sumper M (2007). Putative spermine synthases from *Thalassiosira pseudonana *and *Arabidopsis thaliana *synthesize thermospermine rather than spermine.. FEBS Lett.

[B39] Kröger N, Deutzmann R, Sumper M (1999). Polycationic peptides from diatom biosilica that direct silica nanosphere formation.. Science.

[B40] Kröger N, Deutzmann R, Bergsdorf C, Sumper M (2000). Species-specific polyamines from diatoms control silica morphology.. Proc Natl Acad Sci USA.

[B41] Lenoci L, Camp PJ (2006). Self-assembly of peptide scaffolds in biosilica formation: computer simulations of a coarse-grained model.. J Am Chem Soc.

[B42] Lutz K, Gröger C, Sumper M, Brunner E (2005). Biomimetic silica formation: Analysis of phosphate-induced self-assembly of polyamines.. Phys Chem Chem Phys.

[B43] Sumper M, Kröger N (2004). Silica formation in diatoms: the function of long-chain polyamines and silaffins.. J Mater Chem.

[B44] Oliver S, Kuperman A, Coombs N, Lough A, Ozin GA (1995). Lamellar aluminophosphates with surface patterns that mimic diatom and radiolarian microskeletons.. Nature.

[B45] Mann S, Ozin GA (1996). Synthesis of inorganic materials with complex form.. Nature.

[B46] Kröger N, Lorenz S, Brunner E, Sumper M (2002). Self-assembly of highly phosphorylated silaffins and their function in biosilica morphogenesis.. Science.

[B47] Brunner E, Lutz K, Sumper M (2004). Biomimetic synthesis of silica nanospheres depends on the aggregation and phase separation of polyamines in aqueous solution.. Phys Chem Chem Phys.

[B48] Gordon R, Drum RW (1994). The chemical basis for diatom morphogenesis.. Int Rev Cytol.

[B49] Simpson TL, Volcani BE (1981). Silicon and Siliceous Structures in Biological Systems.

[B50] Iler RK (1979). The Chemistry of Silica.

[B51] Mizutani T, Nagase H, Fujiwara N, Ogoshi H (1998). Silicic acid polymerization catalyzed by amines and polyamines.. Bull Chem Soc Jpn.

[B52] Patel CN, Wortham BW, Lines JL, Fetherston JD, Perry RD, Oliveira MA (2006). Polyamines are essential for the formation of plague biofilm.. J Bacteriol.

[B53] Karatan E, Duncan TR, Watnick PI (2005). NspS, a predicted polyamine sensor, mediates activation of *Vibrio cholerae *biofilm formation by norspermidine.. J Bacteriol.

[B54] Urrutia Mera M, Beveridge TJ (1993). Mechanism of silicate binding to the bacterial cell wall in *Bacillus subtilis*.. J Bacteriol.

[B55] Urrutia MM, Beveridge TJ (1994). Formation of fine-grained metal and silicate precipitates on a bacterial surface (*Bacillus subtilis*).. Chem Geol.

[B56] Armbrust EV, Berges JA, Bowler C, Green BR, Martinez D, Putnam NH, Zhou S, Allen AE, Apt KE, Bechner M, Brzezinski MA, Chaal BK, Chiovitti A, Davis AK, Demarest MS, Detter JC, Glavina T, Goodstein D, Hadi MZ, Hellsten U, Hildebrand M, Jenkins BD, Jurka J, Kapitonov VV, Kröger N, Lau WW, Lane TW, Larimer FW, Lippmeier JC, Lucas S (2004). The genome of the diatom *Thalassiosira pseudonana*: ecology, evolution, and metabolism.. Science.

[B57] Bachrach U (2005). Naturally occurring polyamines: interaction with macromolecules.. Curr Protein Pept Sci.

[B58] Kusano T, Yamaguchi K, Berberich T, Takahashi Y (2007). Advances in polyamine research in 2007.. J Plant Res.

[B59] Phoenix VR, Adams DG, Konhauser KO (2000). Cyanobacterial viability during hydrothermal biomineralisation.. Chem Geol.

[B60] Hou S, Saw JH, Lee KS, Freitas TA, Belisle C, Kawarabayasi Y, Donachie SP, Pikina A, Galperin MY, Koonin EV, Makarova KS, Omelchenko MV, Sorokin A, Wolf YI, Li QX, Keum YS, Campbell S, Denery J, Aizawa S, Shibata S, Malahoff A, Alam M (2004). Genome sequence of the deep-sea gamma-proteobacterium *Idiomarina loihiensis *reveals amino acid fermentation as a source of carbon and energy.. Proc Natl Acad Sci USA.

[B61] Gordon D, Abajian C, Green P (1998). Consed: a graphical tool for sequence finishing.. Genome Res.

[B62] Huang X, Madan A (1999). CAP3: A DNA sequence assembly program.. Genome Res.

[B63] Delcher AL, Harmon D, Kasif S, White O, Salzberg SL (1999). Improved microbial gene identification with GLIMMER.. Nucleic Acids Res.

[B64] Altschul SF, Madden TL, Schaffer AA, Zhang J, Zheng Z, Miller W, Lipman DJ (1997). Gapped BLAST and PSI-BLAST - A new generation of protein database search programs.. Nucleic Acids Res.

[B65] Lowe TM, Eddy SR (1997). tRNAscan-SE: a program for improved detection of transfer RNA genes in genomic sequence.. Nucleic Acids Res.

[B66] Koonin EV, Galperin MY (2002). Sequence - Evolution - Function Computational Approaches in Comparative Genomics.

[B67] Wolf YI, Rogozin IB, Grishin NV, Tatusov RL, Koonin EV (2001). Genome trees constructed using five different approaches suggest new major bacterial clades.. BMC Evol Biol.

[B68] Guerreiro N, Redmond JW, Rolfe BG, Djordjevic MA (1997). New *Rhizobium leguminosarum *flavonoid-induced proteins revealed by proteome analysis of differentially displayed proteins.. Mol Plant Microbe Interact.

[B69] Büttner K, Bernhardt J, Scharf C, Schmid R, Mäder U, Eymann C, Antelmann H, Völker A, Völker U, Hecker M (2001). A comprehensive two-dimensional map of cytosolic proteins of *Bacillus subtilis*.. Electrophoresis.

[B70] Candiano G, Bruschi M, Musante L, Santucci L, Ghiggeri GM, Carnemolla B, Orecchia P, Zardi L, Righetti PG (2004). Blue silver: a very sensitive colloidal Coomassie G-250 staining for proteome analysis.. Electrophoresis.

[B71] Shimotohno KW, Iida J, Takizawa N, Endo T (1994). Purification and characterization of arginine amidinohydrolase from *Bacillus brevis *TT02-8.. Biosci Biotechnol Biochem.

[B72] Yamamoto S, Nakao H, Yamasaki K, Takashina K, Suemoto Y, Shinoda S (1988). Activities and properties of putrescine-biosynthetic enzymes in *Vibrio parahaemolyticus*.. Microbiol Immunol.

[B73] Iyer RK, Kim HK, Tsoa RW, Grody WW, Cederbaum SD (2002). Cloning and characterization of human agmatinase.. Mol Genet Metab.

[B74] Lee MJ, Huang CY, Sun YJ, Huang H (2005). Cloning and characterization of spermidine synthase and its implication in polyamine biosynthesis in *Helicobacter pylori *strain 26695.. Protein Expr Purif.

[B75] Zolg W, Ottow JC (1973). Improved thin-layer technique for detection of arginine dihydrolase among the *Pseudomonas *species.. Appl Microbiol.

[B76] Redmond JW, Tseng A (1979). High-pressure liquid chromatographic determination of putrescine, cadaverine, spermidine and spermine.. J Chromatogr.

[B77] Morgan DM (1998). Determination of polyamines as their benzoylated derivatives by HPLC.. Methods Mol Biol.

[B78] Makarova KS, Sorokin AV, Novichkov PS, Wolf YI, Koonin EV (2007). Clusters of orthologous genes for 41 archaeal genomes and implications for evolutionary genomics of archaea.. Biol Direct.

