# Supplementary material for: Encapsulated in silica: genome, proteome and physiology of the thermophilic bacterium Anoxybacillus flavithermus WK1
Source: Genome Biol. 2008 Nov 17;9(11):R161. doi: 10.1186/gb-2008-9-11-r161 (PMC2614493; doi:10.1186/gb-2008-9-11-r161)
Supplement: Additional data file 1 — Figure S1: phylogenetic distribution of the best BLAST hits of A. flavithermus proteins. Figure S2: the tree of the phylum Firmicutes based on similarity of the phyletic patterns in COGs. Figure S3: two-dimensional gels comparing expression of A. flavithermus proteins from cells grown with or without silica. Figure S4: SDS-PAGE analysis of purified recombinant proteins. Figure S5: thin-layer chromatography-based detection of Aflv_0146 and Aflv_1886 reaction products ornithine and agmatine. Figure S6: HPLC chromatographs showing enzymatic activities of expressed Aflv_0024, Aflv_2749, Aflv_1437 and Aflv_2750 proteins. Figure S7: sequence alignment of A. flavithermus agmatinase Aflv_2749 and arginase Aflv_0146 with various agmatinases and arginases. Table S1: examples of paralogous proteins encoded in the genomes of A. flavithermus and five other bacilli. Table S2: presence or absence of certain metabolic pathway genes in the A. flavithermus genome. Table S3: examples of gene gains and losses in Geobacillus/Anoxybacillus lineages. Table S4: A. flavithermus genes that were found to be up- and down-regulated in cells exposed to silica. [file gb-2008-9-11-r161-S1.pdf]

Additional data file 1 - Figures S1-S7

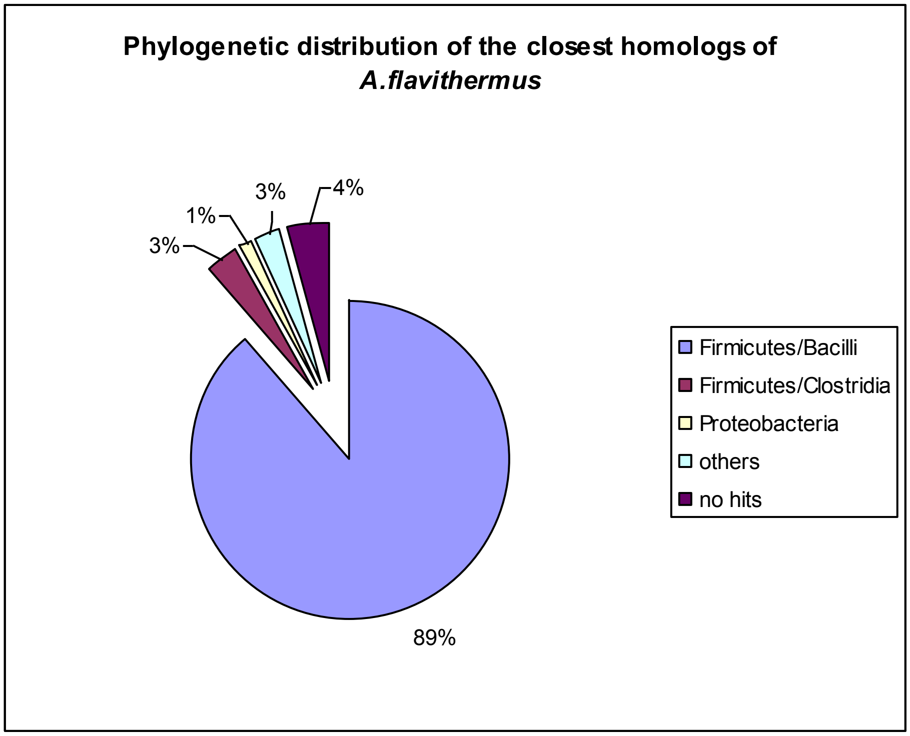

Figure S1 Phylogenetic distribution of the best BLAST hits of *Anoxybacillus flavithermus* proteins. Each sector indicate the fraction of the best BLAST hits of *A. flavithermus* proteins to proteins from the organisms that belong to the class Bacilli (dark blue) or class Clostridia (maroon) within the phylum Firmicutes, to the phylum Proteobacteria (tan), or any other organism (light blue). The purple sector indicates 4% of *A. flavithermus* proteins that have no hits in the current database.

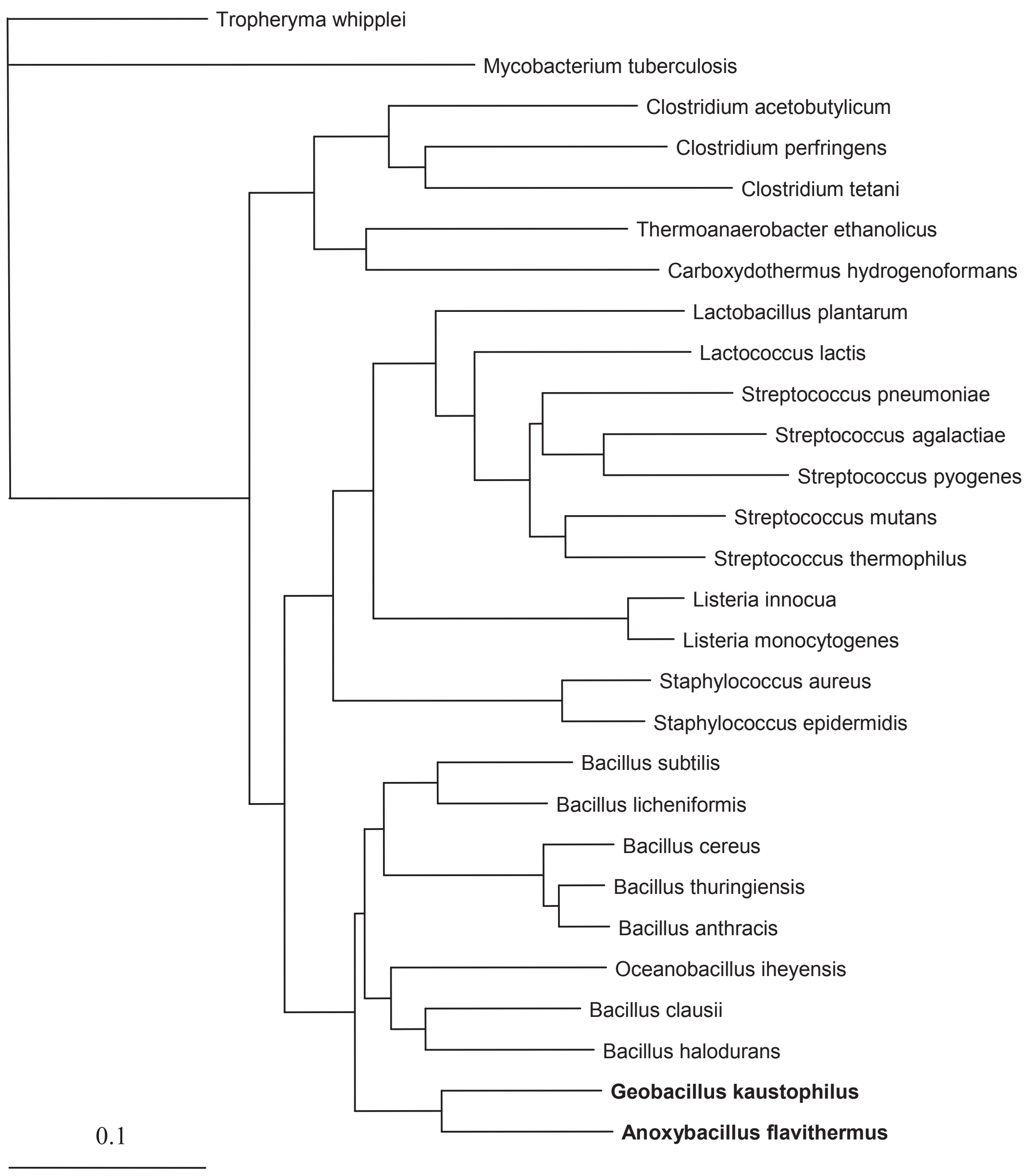

Figure S2 Tree of the phylum *Firmicutes* based on similarity of the phyletic patterns in COGs

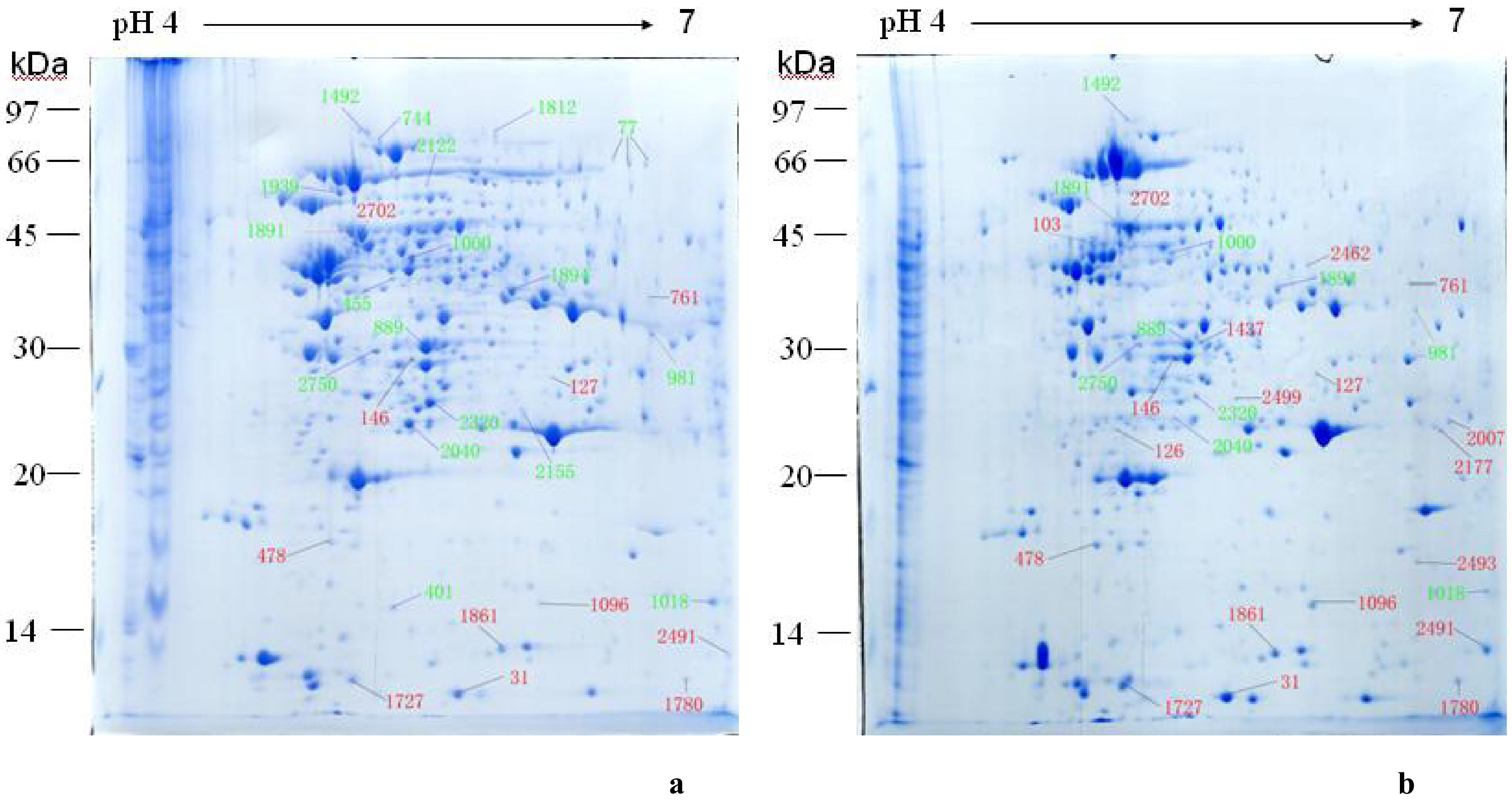

Figure S3 **Comparison of *Anoxybacillus flavithermus* proteins from cells grown with TSB in the absence (a) and presence of silica (b).** Spots identified as up- and down-regulated proteins consistently in three independent experiments are annotated with Aflv gene (locus) numbers as follows: red, proteins up-regulated in the presence of silica; green, proteins down-regulated in the presence of silica.

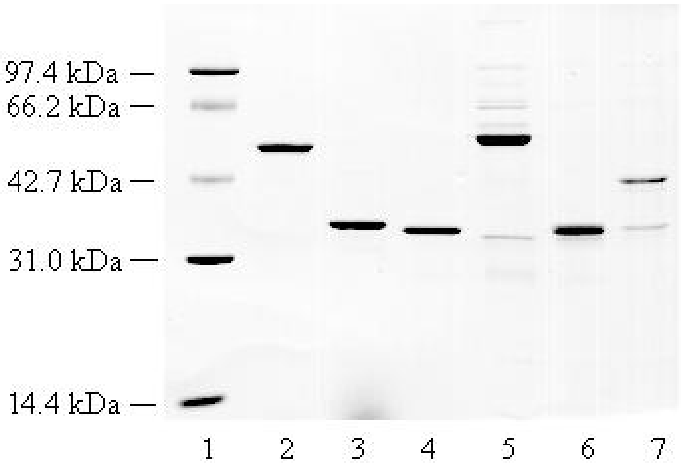

Figure S4 SDS-PAGE analysis of purified recombinant proteins. Lane 1, Mid Range Protein Marker; Lane 2, Aflv_1886 (arginine decarboxylase); Lane 3, Aflv_2749 (agmatinase); Lane 4, Aflv_0146 (arginase); Lane 5, Aflv_0024 (arginine decarboxylase); Lane 6, Aflv_1437 (spermine synthase); Lane 7, Aflv_2750 (spermidine synthase).

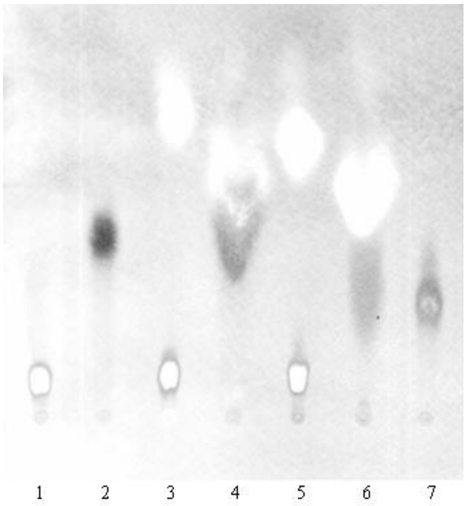

Figure S5 TLC detection of Aflv_0146 and Aflv_1886 reaction products.

Lane 1, standard arginine; lane 2, standard ornithine; lane 3, arginase reaction mixture (containing arginine) without Aflv_0146; lane 4, arginase reaction mixture with Aflv_0146; lane 5, arginine decarboxylase reaction mixture (containing arginine) without Aflv_1886; lane 6, arginine decarboxylase reaction mixture with Aflv_1886; lane 7, standard agmatine. All reaction mixtures were incubated at 37 ^{o}C for 45min.

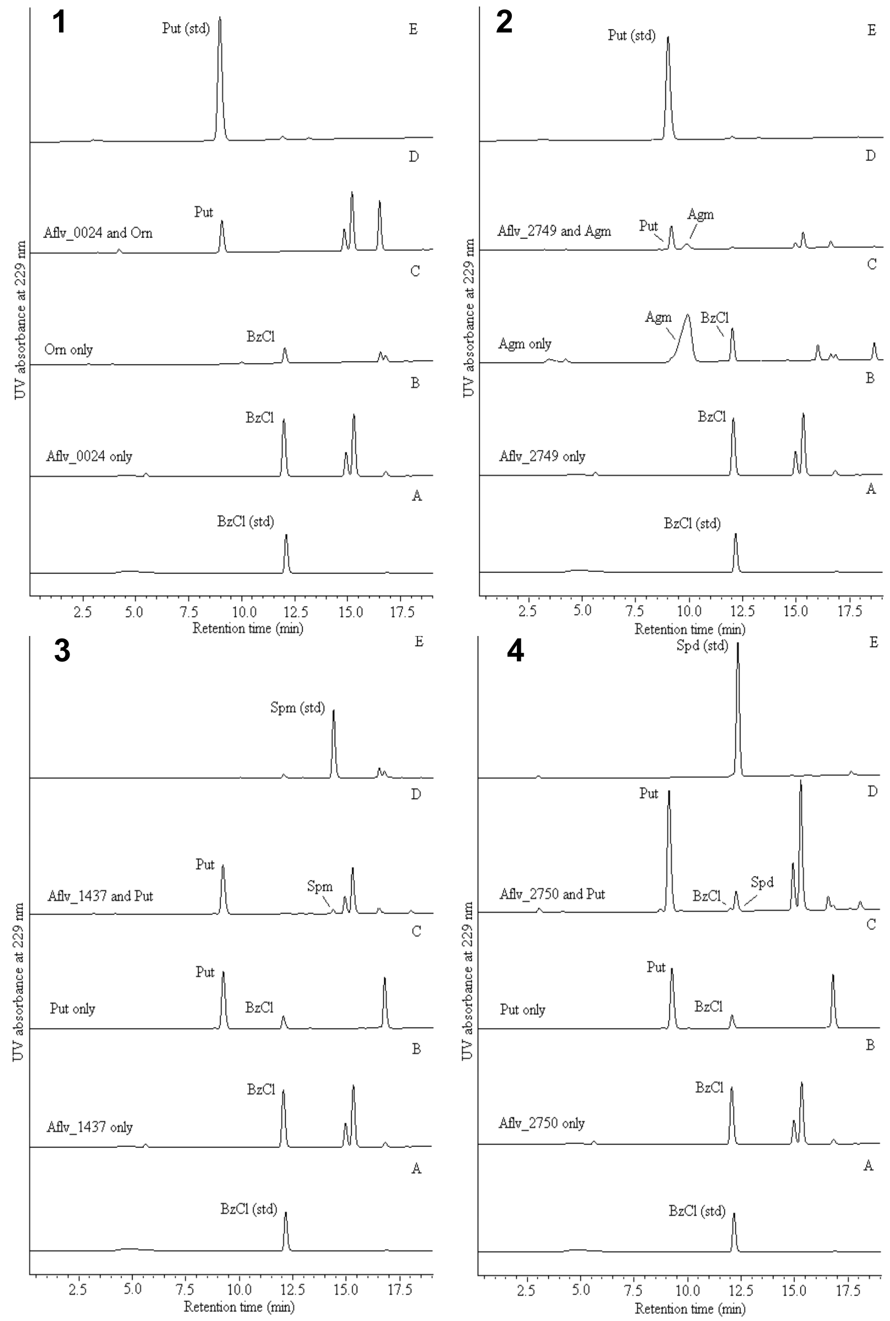

Figure S6 HPLC chromatographs showing conversion of ornithine to putrescine by Aflv_0024 (1), conversion of agmatine to putrescine by Aflv_2749 (2), conversion of putrescine to spermine by Aflv_1437 (3) and conversion of putrescine to spermidine by Aflv_2750 (4). Reaction mixtures were incubated at 37 ^{o}C for 1 hr (panels 1, 2) or 45 ^{o}C for 30 min (panels 3, 4), and derivatized with benzoyl chloride prior to HPLC analysis. All substrate and product peaks were confirmed by MS analysis. Put, putrescine; Agm, agmatine; Spm, spermine; Spd, spermidine; BzCl, benzoyl chloride; std, standard. Ornithine could not be detected under the experimental conditions used.

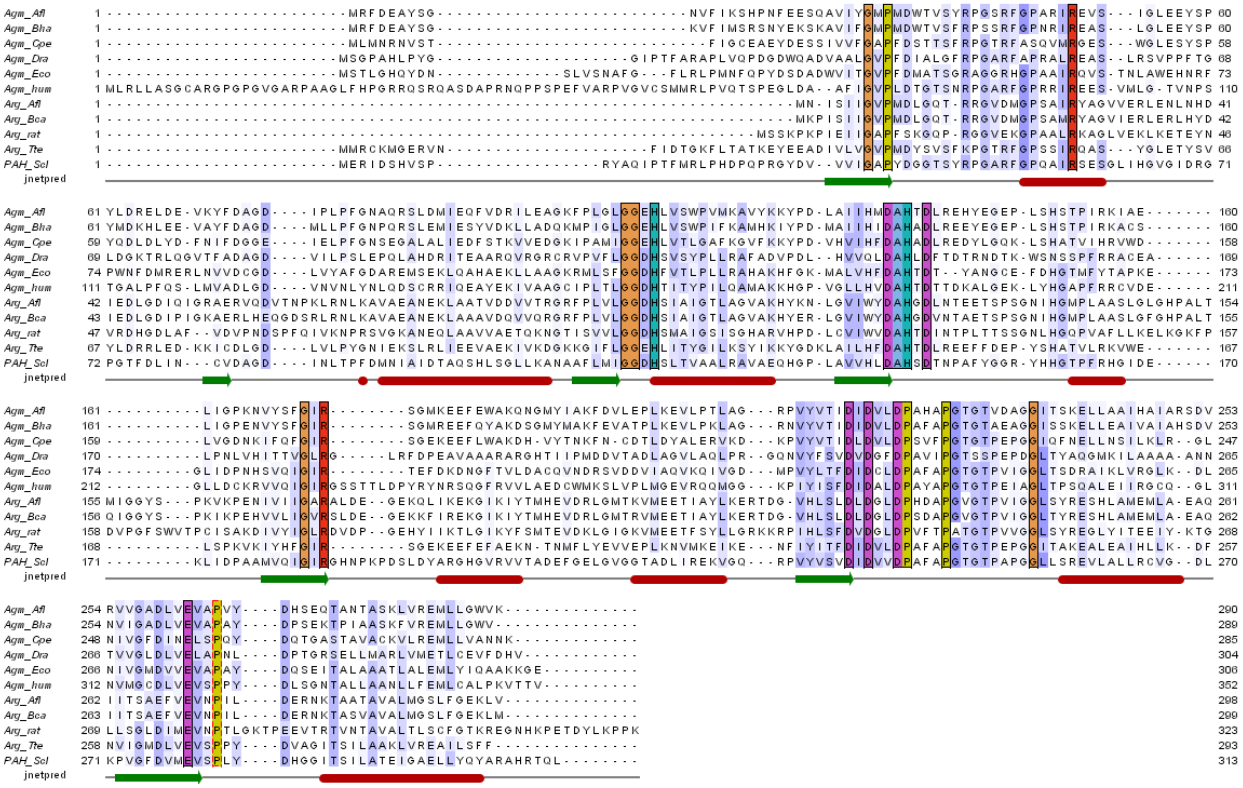

Figure S7 Sequence alignment of *Anoxybacillus flavithermus* agmatinase (Aflv_2749) and arginase (Aflv_0146) with agmatinase from *Deinococcus radiodurans* (PDB: 1WOH) and other agmatinases and arginases. Strictly conserved residues of the ureohydrolase superfamily are highlighted according to the ClustalX color scheme (see Ahn *et al*., 2004, *J. Biol. Chem*. 279:50505-50513, for details).

Additional datra file 1 - Tables S1-S4

Table S1 Paralogous proteins encoded in the genomes of *Anoxybacillus flavithermus* and five other bacilli

A. flav gene	Gene name	Functional annotation	COG no.	Paralogs in *A. flav* genome	Total number of paralogs[a] in the genomes of:				
					B.subt	***B.cere***	***B.halo***	***G.kaus***	***O.ihey***
**Enzymes**									
Aflv_1771	*acpA*	Acyl carrier protein	COG0236	–	3	3	3	2	1
Aflv_2139	*fabF*	3-oxoacyl-(acyl-carrier-protein) synthase II	COG0304	–	11	1	2	5	1
Aflv_2140	*fabH*	3-oxoacyl-[acyl-carrier-protein] synthase III	COG0332	–	2	2	3	2	2
Aflv_0301	*nlpC*	Cell wall-associated amidohydrolase, NlpC/P60 family	COG0791	–	7	3	3	4	4
Aflv_1505	*yjbJ*	Lytic transglycosylase	COG0741	–	5	5	2	7	2
Aflv_1758	*sipT*	Signal peptidase I	COG0681		5	7	2	1	6
Aflv_2287	*prsA*	Parvulin-like peptidyl-prolyl isomerase	COG0760	–	2	4	1	8	3
**Enzyme families**									
Aflv_0990	*yqkF*	Uncharacterized oxidoreductase, aldo/keto reductase family	COG0667	–	5	5	2	2	3
Aflv_2659	*yulF*	Predicted dehydrogenase, MviM family	COG0673	–	8	3	10	2	15
Aflv_2029	*ysfC*	FAD/FMN-containing dehydrogenase, GlcD family	COG0277	–	4	6	3	2	4
Aflv_1432	*yojN*	MoxR-like ATPase	COG0714	–	2	4	2	5	2
Aflv_1072	*ypeB*	Spore cortex hydrolysis protein, HAD superfamily	COG1011	–	2	6	3	3	2
Aflv_2286	*hit*	HIT family hydrolase	COG0537	–	1	2	2	3	2
Aflv_2539	*hprP*	HAD superfamily phosphatase	COG0546	–	1	5	2	3	2
Aflv_0262		NADH:flavin oxidoreductase, nitroreductase family	COG0778	1	7	9	6	5	3
Aflv_0571	*ytcB*	Nucleoside-diphosphate-sugar epimerase	COG0451	1	3	11	6	6	5
Aflv_0194	*ydbR*	DNA or RNA helicase, superfamily II	COG0513	1	4	5	3	4	2
Aflv_1320	*ykwB*	Acetyltransferase (GNAT) family	COG0454	1	29	49	26	2	17
Aflv_1123	*ypjH*	Glycosyltransferase	COG0438	2	9	6	12	15	6
Aflv_0574	*ydaM*	Glycosyltransferase	COG0463	3	14	16	8	17	9
Aflv_2039	*ycgJ*	SAM-dependent methyltransferase	COG0500	7	10	29	16	17	10

**Transcriptional regulators**									
Aflv_1090	*recR*	DNA-binding protein HU (HBsu)	COG0776	–	2	3	2	5	1
Aflv_0736	*yrzC*	Putative transcriptional regulator	COG1959	–	4	5	5	4	3
Aflv_1569	*bofA*	Transcriptional regulator, IclR family	COG1414	–	1	2	3	4	3
Aflv_1234	*gltC*	Transcriptional regulator, LysR family	COG0583	2	19	15	7	8	5
Aflv_0281	*yycF*	DNA-binding response regulator, OmpR family (REC-wHTH domains)	COG0745	4	13	28	16	10	8
**Membrane transport proteins**									
Aflv_2827	–	ABC-type antimicrobial peptide transport system, ATPase component	COG1136	–	7	13	11	7	4
Aflv_2828	–	ABC-type antimicrobial peptide transport system, permease component	COG0577	–	8	19	11	6	4
Aflv_2309	–	ABC-type amino acid transport/signal transduction system, periplasmic component/domain	COG0834	1	7	4	3	7	5
Aflv_1986	–	ABC-type multidrug transport system, permease component	COG0842	–	3	6	6	5	6
Aflv_0207	–	ATPase component of ABC transporter with duplicated ATPase domains	COG0488	–	5	9	3	5	6
Aflv_1589	–	Membrane transporter of cations and cationic drugs	COG2076	–	7	7	4	2	3
Aflv_2851	–	Small-conductance mechanosensitive channel	COG0668	–	3	2	2	4	1
Aflv_1416	–	Permease of the drug/metabolite transporter (DMT) superfamily	COG0697	4	14	20	9	9	2

[a] - The total number of paralogs of each gene in the genomes of *Anoxybacillus flavithermus* strain DSM 2641 (*A.flav*), *Bacillus subtilis* subsp. *subtilis* str. 168 (*B.subt*), *Bacillus cereus* ATCC 10987 (*B.cere*), *Bacillus halodurans* C-125 (*B.halo*), *Geobacillus kaustophilus* HTA426 (*G.kaus*), and *Oceanobacillus iheyensis* HTE831 (*O.ihey*) was calculated as the number of proteins encoded in each of these genomes that were assigned to the given COG (Tatusov *et al*., 2003).

Table S2 Metabolism of *Anoxybacillus flavithermus*

**Metabolic pathway**	**Presence or absence of the pathway genes in the *A. flavithermus* genome, comments**
Purine synthesis and salvage	+
Pyrimidine synthesis and salvage	+
Amino acid biosynthesis	+
Degradation of glycine	+
Glycolysis	+
Gluconeogenesis	Phosphoenolpyruvate synthase/pyruvate phosphate dikinase and fructose-1,6-bisphosphatase are absent. However, PEP synthesis from pyruvate can be catalyzed by a combination of pyruvate carboxylase Aflv_0508 and PEP carboxykinase Aflv_0422. Fructose bisphosphatase could be replaced by a different phosphatase.
Pyruvate oxidation (pyruvate dehydrogenase complex)	+
Pentose phosphate pathway	+<br>Out of 8 enzymes, only the (apparently non-essential) gene coding for 6-phosphogluconolactonase has not been identified
Entner-Doudoroff pathway	–
TCA	+<br>*odhA* and *odhB* genes are lost, replaced by 2-oxoglutarate ferredoxin oxidoreductase (and subunits, Aflv_1523 and Aflv_1524); *bkdAA* (Aflv_0962) and *bkdAB* (Aflv_0963) could also oxidize 2-oxoglutarate
Glyoxylate bypass	+
Mureine biosynthesis	+
Fatty acids biosynthesis	+
B12 (cobalamine) biosynthesis	+
NAD biosynthesis	+
Riboflavin biosynthesis	+
Heme biosynthesis	+
Pantothenate and CoA biosynthesis	+
Menaquinone biosynthesis	+
B_1 (thiamine) biosynthesis	+<br>Thiamine monophosphate kinase gene *thiL* appears to be absent
Folate biosynthesis	+
Biotin biosynthesis	+
B_6 (pyridoxal phosphate) biosynthesis	+
Lipoic acid biosynthesis	+
Deoxyxylylose pathway (isoprenoid biosynthesis)	+
Assimilatory sulfate reduction	+
Mo factor biosynthesis	–
Nitrate/nitrite reduction	–

Table S3 Gene gains and losses in *Geobacillus/Anoxybacillus* lineages

**Selected losses: *Geobacillus/Anoxybacillus***	**Selected gains: *Geobacillus/Anoxybacillus***
Malate/L-lactate dehydrogenase	Cobalamin biosynthesis (15 genes)
Nitrogen regulatory protein PII	Methylmalonyl-CoA mutase
Asparagine synthetase A	Tfp pilus assembly proteins PilE, PilN, PilO
Kynureninase (tryptophan metabolism)	
Arginine deiminase	
Asparagine synthetase A	
H^+/citrate symporter	
Na^+/citrate symporter;	
**Selected losses: *Anoxybacillus***	**Selected gains: *Anoxybacillus***
Molybdenum cofactor biosynthesis	CRISPR system related proteins (4 COGs)
ABC-type molybdate transport system	RecB family exonuclease (2COGs)
Aerobic-type carbon monoxide dehydrogenase (large, middle, small subunits) (present in Geo, molybdopterin dependent)	Predicted site-specific integrase-resolvase
Allophanate hydrolase subunits 1 and 2 (present in Geo)	DNA polymerase elongation subunit (family B)
Urea amidohydrolase (urease) (alpha, beta, gamma subunits) (present in Geo; Geo also gained urease accessory proteins)	Zn-finger domain associated with topoisomerase type I
Glutaminase (present in Geo)	
Urocanate hydratase (present in Geo)	
Histidine ammonia-lyase	
Altronate dehydratase	
Galactokinase	
Galactose-1-phosphate uridyltransferase	
Alpha-galactosidase, family 4 of glycosyl hydrolases	
2-keto-3-deoxy-6-phosphogluconate aldolase	
ABC-type branched-chain amino acid transport system	
Phosphotransferase system cellobiose-specific IIA,B,C components	
TRAP-type C4-dicarboxylate transport system	
Nitrate/nitrite transporter	
Na^+/H^+ antiporter	
Na^+/proline symporter	
Na^+/phosphate symporter	
NhaP-type Na^+/H^+ and K^+/H^+ antiporters	
K^+-transporting ATPase ABC subunits	
Mn^{2+} and Fe^{2+} transporters of the NRAMP family	

Table S4 Genes found to be up- and down-regulated in proteomics experiments

Gene	Gene name	Description	After 8 hours in silica (fold changes in 3 independent experiments)[a]	After 7 days in silica (fold change)
Aflv_0031	*abrB*	Transcriptional regulator AbrB	Up (1.8/2.0/1.6)	Up (1.5)
Aflv_0077	*clpC*	Class III stress response-related ATPase ClpC	**Down (0.2/0/0.4)**	Down (0.4)
Aflv_0103	*tufA*	Translation elongation factor EF-Tu	Up (2.5/2.1/2.0)	Unchanged (0.8)
Aflv_0126	*adk*	Adenylate kinase	Up (2.3/1.9/2.7)	Unchanged (1.1)
Aflv_0127	*map*	Methionine aminopeptidase	Up (2.5/induced/2.2)	Up (1.5)
Aflv_0146	*rocF*	Arginase	**Up (4.0/2.7/1.6)**	Up (induced)
Aflv_0401	*dps*	DNA-binding ferritin-like protein (oxidative damage protectant)	Down (0.3/0/0.3)	Unchanged (1.0)
Aflv_0455	*ampS*	Aminopeptidase	**Down (0.3/0/0.4)**	Up (1.5)
Aflv_0478	*tpx*	Thiol peroxidase	Up (2.4/5.7/1.7)	Up (1.6)
Aflv_0744	*alaS*	Alanyl-tRNA synthetase	Down (0/0/0)	Not detected
Aflv_0761	*metC*	Cystathionine gamma-synthase (O-succinylhomoserine (thiol)-lyase)	Up (4.0/2.9/2.5)	Unchanged (1.1)
Aflv_0889	*ldh*	L-lactate dehydrogenase	Down (0.5/0.4/0.6)	Unchanged (1.0)
Aflv_0981	*yqjM*	NADH:flavin oxidoreductase, Old Yellow Enzyme family	**Down (0.3/0/0.6)**	Up (1.7)
Aflv_1000	*pdp*	Pyrimidine-nucleoside phosphorylase	**Down (0.5/0.2/0.4)**	Not detected
Aflv_1018	*ribH*	Riboflavin synthase beta-chain	**Down (0.4/0.1/0.3)**	Down (0.4)
Aflv_1096	*ndk*	Nucleoside diphosphate kinase	**Up (3.2/4.1/8.4)**	Up (1.9)
Aflv_1437	*speE-like*	Spermidine synthase	**Up (3.3/induced/1.6)**	Not detected
Aflv_1492	*citB*	Aconitase A	Down (0.4/0.2/0.4)	Up (2.1)
Aflv_1727	*cheY*	Chemotaxis response regulator CheY	Up (2.4/1.7/1.9)	Up (1.7)
Aflv_1780	*yloU*	Alkaline shock protein Asp23	**Up (2.4/3.7/2.0)**	Down (0.6)
Aflv_1812	*ileS*	Isoleucyl-tRNA synthetase	**Down (0/0/0)**	Unchanged (1.3)
Aflv_1861	*ylbA*	Uncharacterized conserved protein, YugN family	Up (4.3/1.5/2.4)	Up (1.6)
Aflv_1891	*pdhD*	Pyruvate/2-oxoglutarate dehydrogenase complex, dihydrolipoamide dehydrogenase (E3) component	Down (0.3/0.3/0.4)	Down (0.7)
Aflv_1894	*pdhA*	Pyruvate/2-oxoglutarate dehydrogenase complex, dehydrogenase (E1) component, alpha subunit	Down (0.3/0.5/0.4)	Unchanged (1.4)
Aflv_1939	*ptsI*	Phosphotransferase system (PTS) enzyme I	**Down (0.2/0/0.4)**	Down (0.5)
Aflv_2007	*ykgG*	Uncharacterized conserved protein	**Up (4.1/induced/1.8)**	Not detected
Aflv_2040	*yvaB*	Acyl carrier protein phosphodiesterase	**Down (0.4/0.1/0.5)**	Unchanged (1.0)
Aflv_2122	*yjbG*	Oligoendopeptidase F	Down (0.3/0/0.4)	Down (0.7)
Aflv_2155	*yitV*	Hydrolase of the alpha/beta superfamily	**Down (0.3/0/0.3)**	Up (2.6)
Aflv_2177	*cbiC*	Precorrin isomerase	Up (2.5/induced/1.5)	Unchanged (1.2)
Aflv_2320	*tenA*	Transcriptional activator TenA	Down (<0.5/0.1/0.4)	Not detected
Aflv_2462	*csd*	Cysteine desulfurase	**Up (induced/induced/1.6)**	Unchanged (0.9)
Aflv_2491	*isdI*	Heme-degrading monooxygenase	**Up (8.4/5.6/induced)**	Not detected
Aflv_2493	*hugZ*	Uncharacterized protein, FMN-binding split barrel superfamily	**Up (induced/induced/ induced)**	Not detected
Aflv_2499	*yvaK*	Carboxylesterase (heat shock-induced sigmaB-dependent)	**Up (2.5/induced/1.5)**	Not detected
Aflv_2702	*atpD*	F_0F_1-type ATP synthase, beta subunit	Up (2.6/1.8/7.6)	Unchanged (1.1)
Aflv_2750	*speE*	Spermidine synthase	**Down (0.4/0.6/0.3)**	Unchanged (1.3)

[a] - The data reflect fold changes in the intensity of the staining of protein spots on the 2D gels (Figure S3) with samples from silica-exposed cells as compared to the control cells. Numbers separated with a slash represent fold changes from three independent experiments. Absence of a protein spot on the gel with silica-exposed sample when the same spot was present in the control sample was recorded as "0". Presence of a protein spot on the gel with silica-exposed sample when the same spot was absent in the control sample was recorded as "induced". Significant changes in gene expression (T-test p-value < 0.05) are indicated with bold typeface.
